# Cohesin Releases DNA through Asymmetric ATPase-Driven Ring Opening

**DOI:** 10.1016/j.molcel.2016.01.025

**Published:** 2016-02-18

**Authors:** Ahmed M.O. Elbatsh, Judith H.I. Haarhuis, Naomi Petela, Christophe Chapard, Alexander Fish, Patrick H. Celie, Magda Stadnik, Dejan Ristic, Claire Wyman, René H. Medema, Kim Nasmyth, Benjamin D. Rowland

**Affiliations:** 1 Division of Cell Biology, The Netherlands Cancer Institute, Plesmanlaan 121, 1066 CX Amsterdam, The Netherlands; 2 Department of Biochemistry, University of Oxford, South Parks Road, OX1 3QU, Oxford, United Kingdom; 3 Division of Biochemistry, The Netherlands Cancer Institute, Plesmanlaan 121, 1066 CX Amsterdam, The Netherlands; 4 Department of Genetics, Cancer Genomics Netherlands, and Department of Radiation Oncology, Erasmus University Medical Center, 3000 CA Rotterdam, The Netherlands

## Abstract

Cohesin stably holds together the sister chromatids from S phase until mitosis. To do so, cohesin must be protected against its cellular antagonist Wapl. Eco1 acetylates cohesin’s Smc3 subunit, which locks together the sister DNAs. We used yeast genetics to dissect how Wapl drives cohesin from chromatin and identified mutants of cohesin that are impaired in ATPase activity but remarkably confer robust cohesion that bypasses the need for the cohesin protectors Eco1 in yeast and Sororin in human cells. We uncover a functional asymmetry within the heart of cohesin’s highly conserved ABC-like ATPase machinery and find that both ATPase sites contribute to DNA loading, whereas DNA release is controlled specifically by one site. We propose that Smc3 acetylation locks cohesin rings around the sister chromatids by counteracting an activity associated with one of cohesin’s two ATPase sites.

## Highlights

• Cohesin’s DNA release involves an asymmetric activity within its ATPase machinery
• DNA release is driven by the ATPase site proximal to the locking acetylation marks
• Cohesin’s DNA release is ATPase driven in yeast and humans
• Cohesin’s loading onto DNA is controlled by both of its ATPase sites

## Graphical Abstract

**Figure:**
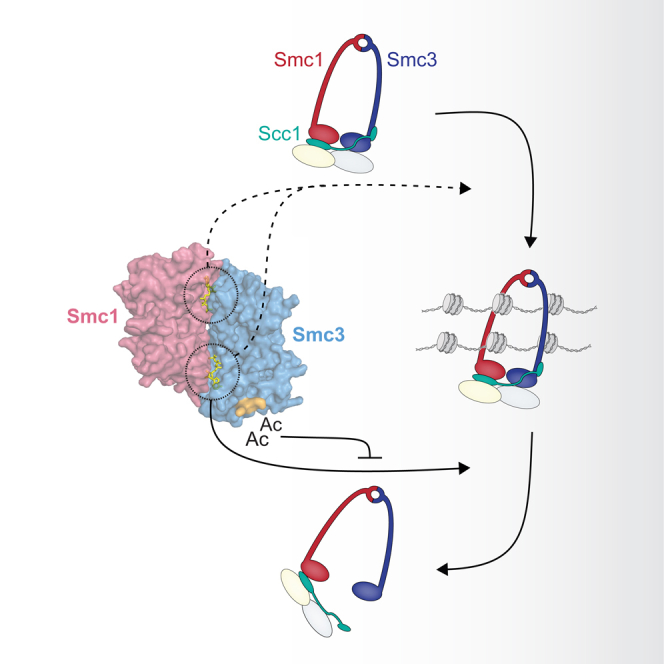

## Teaser

Tight regulation of DNA entrapment and release by the cohesin complex is crucial for its multiple cellular functions. Elbatsh et al. find that cohesin’s release from DNA requires an activity associated with one of its ATPase sites, whereas both sites control cohesin’s loading onto DNA.

## Introduction

Faithful chromosome segregation in mitosis is essential for genomic stability. This process is highly dependent on the cohesin complex, which holds together the sister chromatids of each chromosome. By resisting the pulling forces of microtubules up to the moment that all chromosomes are correctly attached, cohesin ensures that the sister chromatids separate to the opposite poles of the cell and that each of the daughter cells receives an equal karyotype (Nasmyth and Haering, 2009, Peters et al., 2008).

The cohesin complex consists of three core components (Smc1, Smc3, and Scc1) that together constitute a huge tri-partite ring. Smc1 and Smc3 each have head domains, which together form a composite ABC-like ATPase, and have a second shared interface at the other end of their 50-nm-long coiled coils that is referred to as the hinge. Scc1 in turn bridges the head domains of both Smc proteins (Gruber et al., 2003, Haering et al., 2002). The complex also has two additional subunits, Scc3 and Pds5, with regulatory functions (Haarhuis et al., 2014). Cohesin is thought to hold together the sister chromatids by co-entrapping them inside its ring-shaped structure (Haering et al., 2008).

Cohesin stably holds together the sister chromatids from DNA replication until anaphase onset. When cohesin rings are not in their cohesive state, they have a transient association with chromatin that appears to be the consequence of a continuous cycle of DNA entrapment and release (Eichinger et al., 2013, Gerlich et al., 2006). DNA entrapment by cohesin is dependent on the Scc2/Scc4 loader complex (Ciosk et al., 2000, Murayama and Uhlmann, 2014), while release involves cohesin’s antagonist Wapl (Gandhi et al., 2006, Kueng et al., 2006).

Scc2/Scc4 stimulates ATP hydrolysis by cohesin’s ATPase domain, but how this may regulate the entrapment of DNA is unknown (Murayama and Uhlmann, 2014). Cohesin’s ATPase domain is its best-conserved domain, but our molecular understanding of its role is limited. This region sandwiches two ATPs between the head domains of Smc1 and Smc3, and ATP hydrolysis is necessary for the stable association of cohesin with chromatin (Arumugam et al., 2003, Arumugam et al., 2006, Hu et al., 2011, Ladurner et al., 2014, Murayama and Uhlmann, 2014, Weitzer et al., 2003).

Cohesin’s removal factor Wapl binds to Pds5 and Scc3 (Gandhi et al., 2006, Hara et al., 2014, Kueng et al., 2006, Ouyang et al., 2013, Rowland et al., 2009, Shintomi and Hirano, 2009) and can bind to Smc3’s ATPase domain (Chatterjee et al., 2013), but how these interactions affect cohesin’s release from DNA is unknown. Cohesin has a distinct DNA exit gate that lies at the interface connecting Smc3’s ATPase domain and the N terminus of Scc1 (Buheitel and Stemmann, 2013, Chan et al., 2012, Eichinger et al., 2013). Recent work shows that the N terminus of Scc1 binds to the coiled coil just above the ATPase domain of Smc3 (Gligoris et al., 2014, Huis in ’t Veld et al., 2014). The C terminus of Scc1 however binds in a very different manner to the bottom of Smc1’s ATPase domain (Haering et al., 2004).

**Figure 1:**
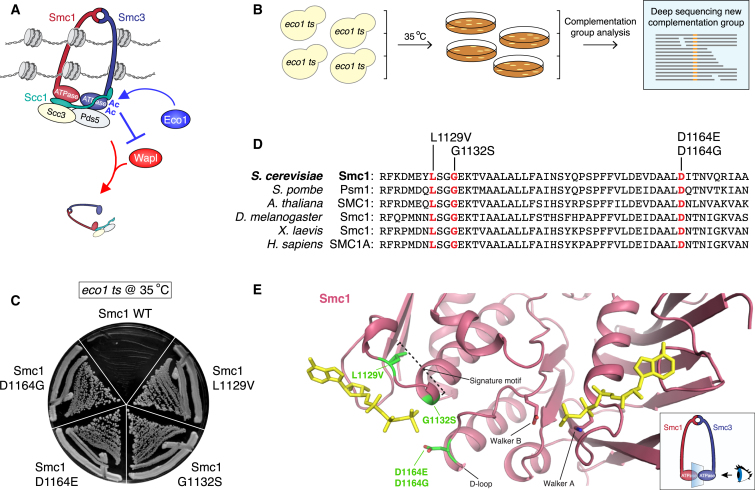
Mutations in Smc1’s ATPase Domain Bypass the Need for Budding Yeast Eco1 (A) Cohesin transiently associates with DNA due to Wapl-mediated cohesin removal activity. In S phase, Eco1-dependent Smc3 acetylation locks cohesin rings and renders them resistant to Wapl. These cohesin rings stably hold together the sister DNAs until mitosis. (B) Schematic representation of *eco1 ts* suppressor screen. (C) Mutations in Smc1’s ATPase domain rescue the lethality of *eco1 ts* at the non-permissive temperature (streaks clockwise, from top: K16297: *eco1-1*; BR348: *SMC1 L1129V*, *eco1-1*; BR448: *SMC1 G1132S*, *eco1-1*; BR355: *SMC1 D1164E*, *eco1-1*; BR363: *SMC1 D1164G*, *eco1-1*). (D) Mutated residues in Smc1’s ATPase domain are conserved from yeast to humans. The mutated residues are indicated in red. Amino acid numbers correspond to the *S. cerevisiae* protein. (E) Mutated residues are located in the signature motif (LSGGE) and D-loop of Smc1’s ATPase domain. Structure of the ATPase domain of Smc1 (PDB: 1W1W) visualized from the angle of Smc3’s ATPase domain (see inset). The mutated residues are shown in green. See also Figure S1.

From S phase onward, cohesin stably holds together the sister chromatids. To achieve this, cohesin must be protected against Wapl-mediated removal activity. This protection is provided by the essential acetyltransferase Eco1, which acetylates cohesin’s Smc3 subunit at two highly conserved lysines that are located on the outside part of its ATPase domain (Rolef Ben-Shahar et al., 2008, Rowland et al., 2009, Unal et al., 2008, Zhang et al., 2008). This acetylation in essence acts as a lock, as it renders cohesin resistant to Wapl (Chan et al., 2012, Lopez-Serra et al., 2013) (Figure 1A).

Cohesin and virtually all of its regulators are conserved from yeast to humans. The notable exception is Sororin, which only appears to exist in animal cells. Sororin is recruited to acetylated cohesin complexes and is essential for the protection against Wapl (Lafont et al., 2010, Nishiyama et al., 2010). How Smc3 acetylation and Sororin render cohesin resistant against Wapl is largely unknown. And how Wapl in fact drives cohesin from chromatin remains a mystery. We performed an unbiased genetic screen in budding yeast to learn about the mechanism of Wapl-mediated cohesin removal. Hereby we identified an unexpected asymmetric activity within the heart of cohesin’s ATPase machinery that drives DNA release from cohesin rings. We find that this key mechanism is conserved from yeast to humans.

## Results

### A Crucial Role for Smc1’s ATPase Domain in Cohesin’s Release from DNA

From S phase until mitosis, cohesin rings are protected against Wapl. A key regulator of this protection is the Eco1 acetyltransferase that through the acetylation of Smc3 renders cohesin refractory to Wapl. In order to learn about the mechanism by which Wapl drives cohesin from chromatin, we performed a genetic screen in budding yeast for mutants that bypass the need for Eco1. We chose this system, as we and others previously showed that Wapl deletion supports viability in the absence of Eco1 (Rolef Ben-Shahar et al., 2008, Rowland et al., 2009, Sutani et al., 2009, Unal et al., 2008). This screening method allows for the unbiased identification, with amino acid resolution, of protein domains that are essential for Wapl-mediated cohesin removal (Rolef Ben-Shahar et al., 2008, Rowland et al., 2009, Sutani et al., 2009).

This system entails the large-scale screening for spontaneous suppressors using a temperature-sensitive *eco1-1* yeast strain. In order to find mutant alleles of genes that were not previously implicated in Wapl-mediated cohesin release, we scaled up our screening to include 500 independent parental *eco1-1* strains. We isolated no more than one suppressor at the non-permissive temperature of each parental strain, which we followed up with complementation-group analysis. Using this method, we isolated a complementation group that had no mutations in *WAPL*, *SMC3*, *SCC3*, or *PDS5*. We identified the mutations that apparently bypassed the need for Eco1 by full-genome sequencing (Figures 1B and 1C). Intriguingly, these mutations clustered in a small patch at the heart of the ABC-like ATPase domain of Smc1. Two of the mutations (L1129V and G1132S) affect the Signature motif (LSGGE) and two (D1164E and D1164G) alter the same key amino acid in the so-called D-loop (Figures 1E and S1).

These mutations are exciting for a number of reasons. First, they pinpoint a crucial role of cohesin’s ATPase domain. The current understanding is that ATP hydrolysis is somehow required for the stable association of cohesin with chromatin (Arumugam et al., 2003, Arumugam et al., 2006, Hu et al., 2011, Ladurner et al., 2014, Murayama and Uhlmann, 2014, Weitzer et al., 2003). Our results indicate that this ATPase domain is actually required for cohesin’s removal from DNA. In addition, the mutated amino acids are conserved through all eukaryotes analyzed, suggesting that they are important for a highly conserved function of the cohesin complex (Figure 1D).

### Smc1 ATPase Mutants Allow Viability of Budding Yeast in the Total Absence of Eco1

**Figure 2:**
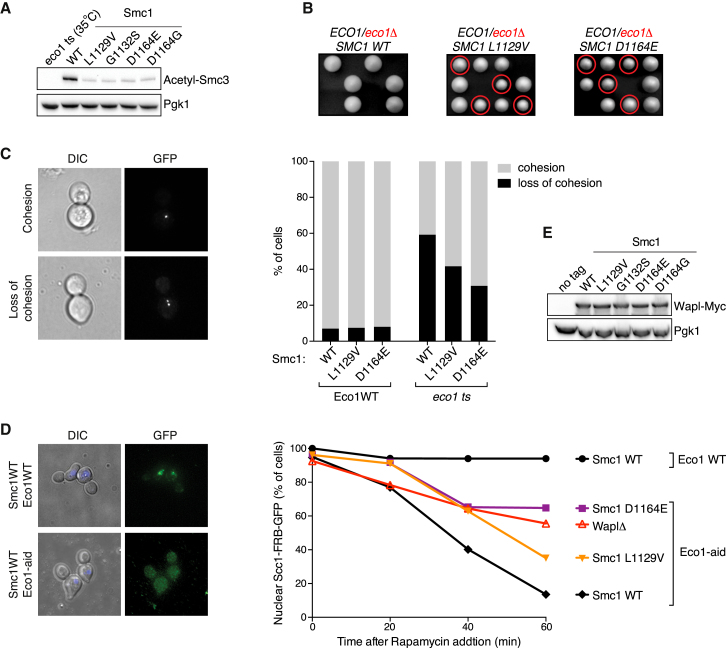
Mutations in Smc1’s ATPase Domain Allow Cohesion and Stable Chromatin Association without Eco1 (A) Western blot analysis of Smc3 acetylation comparing Smc1 ATPase mutants to wild-type (K16297: *eco1-1*; BR645: *SMC1 Wild-type*; BR625: *SMC1 L1129V*; BR643: *SMC1 G1132S*; BR627: *SMC1 D1164E*; BR629: *SMC1 D1164G*). Cells were grown at 37°C prior to harvesting in order to inactivate Eco1. (B) Representative Smc1 mutations allow viability of yeast in the total absence of Eco1. Tetrad dissection of heterozygous ECO1/*eco1*Δ strains in either a wild-type background or in a background heterozygous for mutants Smc1 L1129V (BR463) or Smc1 D1164E (BR464). The spores that harbor the *eco1* deletion marker are encircled. Three representative dissections are shown out of at least 40 per genotype. (C) Representative mutations in the Smc1 ATPase domain support good cohesion and partially rescue the cohesion defect of a temperature sensitive *eco1-1* strain at the non-permissive temperature. Percentage of cells with cohesed or separated GFP dots marking the *URA3* locus in wild-type Smc1 (BR455: *ECO1* and BR426: *eco1-1*) or mutant Smc1 L1129V (BR459: *ECO1* and BR428: *eco1-1*) and Smc1 D1164E (BR461: *ECO1* and BR429: *eco1-1*) yeast. Cells were synchronized in G1 and released at the non-permissive temperature. Cohesion was scored in metaphase-arrested cells. Images depict examples of cells with cohesion (above) and loss of cohesion (below). (D) Representative mutations in the Smc1 ATPase domain allow stable chromatin association in the absence of Eco1. Yeast were synchronized in G1 and released in the presence of synthetic auxin to inactivate Eco1-aid. Cells were arrested in nocodazole and Scc1-FRB-GFP was anchored away upon addition of Rapamycin (BR439: *Wild-Type*; BR431: *ECO1-AID*, BR433: *WPL1Δ*, *ECO1-AID*, BR445: *SMC1 D1164E*, *ECO1-AID* and BR572: *SMC1 L1129V*, *ECO1-AID*). Images depict examples of cells with nuclear retention of Scc1-FRB-GFP (above) or with loss of nuclear retention (below). (E) Wapl levels are unaffected in Smc1 ATPase mutant cells. Asynchronously growing cells expressing Myc-tagged Wapl were analyzed by western blot (K699: *Wild-Type No tag*; K15721: *SMC1 WT*, BR651: *SMC1 L1129V*, BR653: *SMC1 G1132S*, BR655: *SMC1 D1164E* and BR657: *SMC1 D1164G*). Pgk1 acts as a loading control.

Smc3 acetylation was recently shown to be dependent on cohesin-mediated ATP hydrolysis (Ladurner et al., 2014). As these mutations are located in Smc1’s ATPase domain, we tested whether they affected Smc3 acetylation using an acetyl-Smc3-specific antibody. Interestingly, the Smc1 mutations all partially impaired Smc3 acetylation (Figure 2A). This indicates that these mutants survive with reduced Smc3 acetylation.

We went on to test whether the Smc1 mutants can even support viability of budding yeast in the total absence of Eco1. To this end, we crossed representative strains that harbored an Smc1 mutant from either domain (the Signature motif mutant L1129V and the D-loop mutant D1164E) with a wild-type strain. In each of the resulting diploid strains we deleted one of the two *ECO1* alleles and then triggered the strains to go through meiosis. The four haploid spores were separated by tetrad dissection. As *ECO1* is an essential gene, control strains never had more than two viable spores. Importantly, when the diploids harbored either the L1129V or the D1164E mutation, we frequently found three or four viable spores. Subsequent genotyping of these spores showed that each of these mutants indeed allowed spore viability in the total absence of Eco1 (Figure 2B).

### Smc1 ATPase Mutants Allow Cohesion and Stable DNA Binding in the Absence of Eco1

We then tested whether the mutants allowed cohesion in the absence of Eco1. For this we used a so-called GFP dot assay (Michaelis et al., 1997). We used haploid yeast strains in which the *URA3* locus is marked by a GFP dot. Upon DNA replication this sequence is replicated and the sister chromatids are held together in an Eco1-dependent manner. Eco1 inactivation prior to S phase, using a temperature-sensitive allele, indeed displayed loss of cohesion in metaphase-arrested cells. Both representative Smc1 mutations L1129V and D1164E however partially rescued this cohesion defect (Figures 2C and S2). Notably, neither Smc1 mutant displayed an overt cohesion defect in a wild-type Eco1 background in this assay.

Next, we tested whether the Smc1 mutants allowed stable binding of cohesin to DNA without Eco1. We made use of a recently developed system (Lopez-Serra et al., 2013) that is based upon the Anchor Away technique (Haruki et al., 2008). The strains harbor Scc1 with a FRB-GFP tag. Upon the addition of Rapamycin, Scc1-FRB-GFP is shuttled out of the nucleus by the RPL13A-FKBP12 fusion, unless it is stably bound to DNA. Eco1, through acetylation of Smc3, locks cohesin rings on the DNA and renders them resistant to Wapl. The inactivation of Eco1 before S phase entry (using an Auxin-inducible degron) prevented stable DNA binding and allowed the shuttling of Scc1-FRB-GFP to the cytoplasm in a manner that was largely Wapl dependent. We found that both Smc1 ATPase mutants allowed stable DNA binding in the absence of Eco1 to a degree that was similar to inactivation of Wapl (Figure 2D). We then ensured that none of the Smc1 ATPase mutants affected Wapl expression levels (Figure 2E). Together, these results indicate that these key amino acids in Smc1’s ATPase domain are required for cohesin’s release from DNA.

### Smc1 ATPase Mutants Are Severely Impaired in ATP Hydrolysis

The Smc1 mutations L1129V and G1132S both affect the Signature motif, which is an integral part of the ATP binding pocket and is important for ATPase head engagement. The D1164E and D1164G mutants both affect the D-loop. This loop is thought to be important for the correct alignment of the water molecule required for the hydrolysis reaction (Procko et al., 2009). In order to dissect which part of the ATPase cycle is affected by these mutations, we performed a set of biochemical assays.

**Figure 3:**
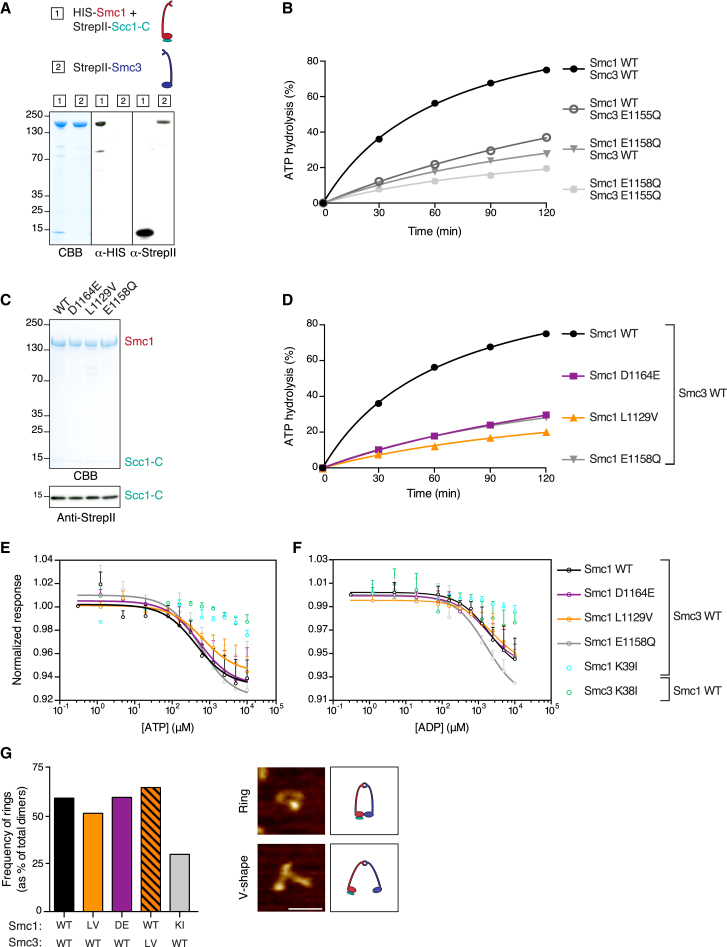
Smc1 ATPase Mutants Are Severely Impaired in ATP Hydrolysis (A) Recombinant expression of cohesin subunits. Coomassie brilliant blue staining (CBB) and western blots of full-length HIS_6_-Smc1 and the co-expressed C terminus of Scc1 (StrepII-Scc1-C). Full-length StrepII-Smc3 was expressed alone. Cartoons depict the cohesin subunits. (B) Time course analysis of ATP hydrolysis using either wild-type or the depicted cohesin mutants. ATP hydrolysis of radiolabelled ATP was measured by thin-layer chromatography. All Smc1 proteins were co-purified with Scc1-C. Depicted is a representative experiment from at least three independent protein purifications of each mutant. (C) SDS-PAGE, Coomassie brilliant blue staining (CBB), and western blot analysis of representative preps of either wild-type or mutant Smc1 co-purified with Scc1-C. (D) Experiment performed as in (B) but using the depicted cohesin mutants. (E) Microscale thermophoresis (MST) binding curves of ATP titrated and co-incubated with the depicted fluorescently labeled Smc1 and Smc3 mutants. Error bars show SEM of two independent experiments. All Smc1 proteins were co-purified with Scc1-C. See also Table S1. (F) As in (E) but with titrated ADP. See also Table S1. (G) Analysis of full-length Smc heterodimers by scanning force microscopy (SFM). Percentage of heterodimers with a ring-shaped conformation (of total dimers, counted as V-shape or ring shape). At least 70 dimers per condition were quantified (except for Smc1 D1164E n = 27). Representative SFM images are shown on the right. The scale bar represents 50 nm. Color represents height from 0 nm to 2 nm, dark to light.

As the mutations are likely to affect ATP hydrolysis, we wished to first perform in vitro ATPase assays. To this end, we expressed recombinant full-length Smc1 and Smc3 and the C terminus of Scc1 in insect cells and purified these proteins to homogeneity (Figure 3A). This combination of proteins was previously used to successfully measure ATPase activity of the budding yeast cohesin complex (Arumugam et al., 2006). We performed ATPase assays using thin-layer chromatography and radiolabeled ATP. Importantly, our ATPase assays fully recapitulated the previously published ATPase activity for these proteins (Figure 3B) (Arumugam et al., 2006). As controls we used the classical Walker B mutants Smc1 E1158Q and Smc3 E1155Q. Each of these single mutations significantly inhibited ATP hydrolysis, while the combination further reduced hydrolysis. We should note that neither of these mutants supports either viability or cohesion (Arumugam et al., 2003, Hu et al., 2011).

Then we measured the ATPase activity of the Smc1 mutants L1129V and D1164E. Both proteins were expressed to the same level and were equally well purified as the wild-type protein (Figure 3C). To our major surprise, however, these proteins had severely reduced ATPase activity (Figure 3D). Both mutants in fact reduced ATP hydrolysis as much as, if not more than, the Smc1 E1158Q Walker B mutant. This result is highly unexpected. As described above, our L1129V and D1164E Smc1 mutants very well support both cohesion and viability, and they yield complexes that are very stably associated with DNA.

Next, we assessed the binding affinities of the mutants to ATP and ADP using MicroScale Thermophoresis (MST) assays. We included Walker A mutants of Smc1 (K39I) and Smc3 (K38I) that are predicted to be defective in nucleotide binding (Arumugam et al., 2003). Whereas each of the Walker A mutations efficiently abrogated binding to ATP and ADP, we found that the Smc1 L1129V, D1164E and E1158Q mutants all had wild-type-like affinity to both ATP and ADP (Figures 3E, 3F, and S3; Table S1).

Our finding that each of the individual mutants Smc1 K39I and Smc3 K38I prevents ATP binding by an otherwise wild-type Smc heterodimer, shows that nucleotide binding by cohesin is a cooperative event. Apparently neither Smc1 nor Smc3 can stably bind to ATP by itself. The simplest explanation for this result is that Smc1 and Smc3 together stably sandwich both ATPs between their ATPase heads and that binding to both ATPs is required for this head engagement. Because ATP is much smaller than the Smc proteins, it seems likely that the detected changes in thermophoresis of the fluorescently labeled proteins are due to a conformational change induced by the engagement of the Smc heads upon ATP binding.

We should note that both wild-type and mutant cohesin complexes appear to have similar affinities to ADP that all are well beyond physiological concentrations (>1 mM). This indicates that product inhibition due to slow ADP release is unlikely to be a rate-limiting step for cohesin’s ATP hydrolysis in vivo.

As the Smc1 mutants L1129V, D1164E, and E1158Q efficiently bind ATP, this implies that they are presumably all proficient in some form of ATPase head engagement. We further assessed ATPase head engagement using scanning-force microscopy (SFM). We co-incubated full-length Smc1 with full-length Smc3 in the presence of ATP and seeded the samples on Mica surface for SFM analysis. We detected two types of structures (V shapes and ring shapes) that were absent from samples with just separate Smc subunits, which indicates that these structures reflect Smc1/Smc3 heterodimers (Figure 3G). Smc1 and Smc3 tightly bind to each other through their hinge interface. We therefore assume that the V shapes reflect Smc1 and Smc3 heterodimerized at this interface and that the ring shapes depict Smc1 and Smc3 that are simultaneously engaged through their ATPase head domains. As expected, the ring shapes were less abundant in dimers harboring the ATP binding mutant Smc1 K39I. Importantly, we detected a similar ratio of ring structures for the wild-type dimers as for dimers harboring the Smc1 mutants L1129V and D1164E (Figure 3G), again indicating that these mutations do not abrogate head engagement.

Together, these results show that the Smc1 mutants L1129V and D1164E can bind normally to both ATP and ADP, that they apparently can engage their ATPase heads, but that they are impaired in their ability to hydrolyze ATP. The mutations therefore affect either the hydrolysis reaction itself, or they may affect a conformational change that might take place between ATP-dependent head engagement and hydrolysis. This conformational change could then, for example, entail the transition to a certain type of ATPase head engagement that is required for hydrolysis. In both of these scenarios, however, the net result is reduced ATP hydrolysis.

### Cohesin’s Distribution along Chromosomes Is Not Affected by Smc1 ATPase Mutations

Hydrolysis of each of cohesin’s associated ATPs is generally considered to be equally important for cohesion. This assumption is based upon the finding that Walker B mutations in Smc1 (E1158Q) and Smc3 (E1155Q) in essence yield the same result, namely no stable DNA association, no cohesion, and no viability (Arumugam et al., 2003, Hu et al., 2011). This defect is mirrored by a typical distribution of these Walker B mutants on chromosomes. They are found highly enriched at centromeres and to a lesser extent also at other cohesin loading sites, but they are otherwise virtually absent. These Walker B mutants apparently are recruited to the loading sites, but as they can’t entrap DNA, they are thought to be unable to slide along DNA to the surrounding regions (Hu et al., 2011, Hu et al., 2015).

Above, we describe Smc1 mutants that are as hydrolysis deficient as the Walker B mutants but support viability. We therefore tested the effect of the representative Smc1 mutations L1129V and D1164E on cohesin’s binding to chromosomes. We used a recently developed technique called calibrated ChIP-seq (Hu et al., 2015). This method allows the accurate genome-wide comparison of both the abundance and the distribution of cohesin on chromosomes between different yeast strains. We performed calibrated ChIP-seq on Scc1-PK expressed in yeast that harbored wild-type Smc1 or either of the Smc1 ATPase mutants L1129V and D1164E.

**Figure 4:**
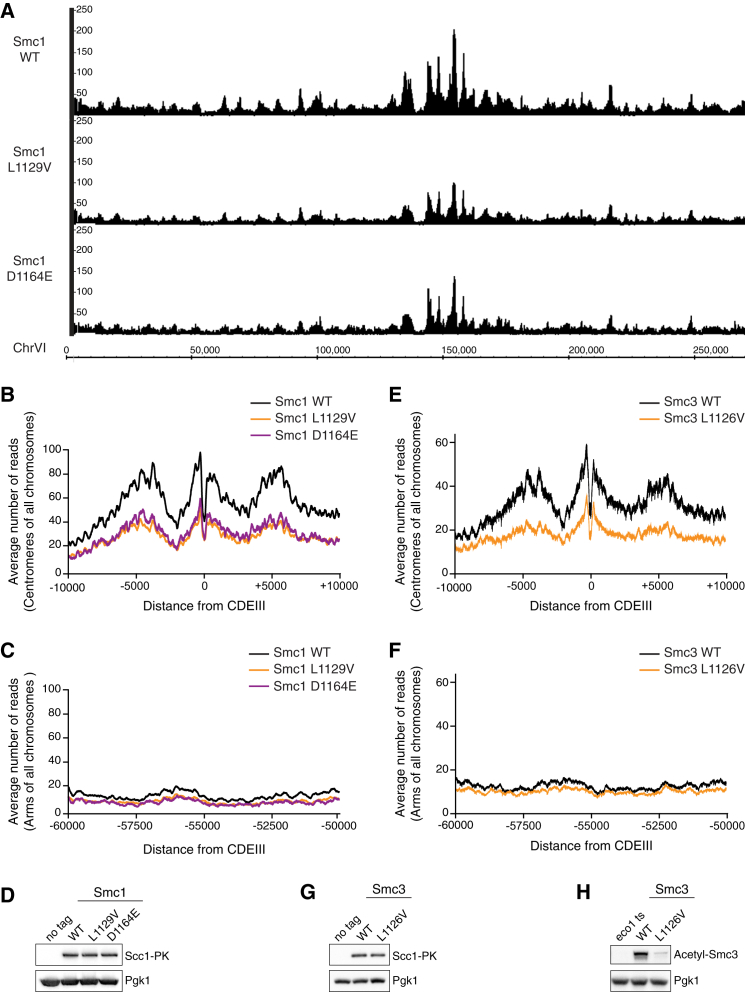
Cohesin’s Distribution along Chromosomes Is Not Affected by Smc1 ATPase Mutations (A) Calibrated ChIP-seq profiles show similar Scc1-PK distribution along chromosome VI. Extracts prepared from a mixture of exponentially grown *C. glabrata* (K23308) and *S. cerevisiae* cells harboring wild-type (BR645), mutant Smc1 L1129V (BR625), or D1164E (BR627) were processed for calibrated ChIP-seq. The y axis indicates the number of reads covering every base pair and the x axis indicates the position of every base pair adopted from SGD (http://www.yeastgenome.org). (B) Scc1-PK distribution at centromeric regions in cells with mutant Smc1 L1129V and D1164E is similar to wild-type, but reduced by approximately 40%. Experiment performed as in (A). The plot depicts the average distribution of cohesin around the centromere (CDEIII) of all chromosomes. See also Figure S4A. (C) Scc1-PK distribution at arm regions in cells with mutant Smc1 L1129V and D1164E is similar to wild-type, but reduced by about 20%. Experiment performed as in (A). The plot depicts the average distribution of Scc1-PK at arm regions spanning from 60 to 50 kb from the centromere (CDEIII) of all chromosomes. See also Figure S4B. (D) Expression levels of Scc1-PK are similar in control and Smc1 L1129V and D1164E cells (K699: *Wild-Type No tag*; BR645: *SMC1 WT*; BR625: *SMC1 L1129V* and BR627: *SMC1 D1164E*). (E) As in (B) but with Smc3 L1126V mutant cells (BR776: SMC3 WT; BR777: SMC3 L1126V). Expression of endogenous Smc3 under the control of a galactose-inducible promoter was suppressed on glucose. See also Figure S4C. (F) Scc1-PK distribution at arm regions in cells with mutant Smc3 L1126V is similar to wild-type, but reduced by about 20%, like Smc1 mutants. Plot is as in (C). See also Figure S4D. (G) Expression levels of Scc1-PK are similar between control and Smc3 L1126V cells (K699: *Wild-Type No tag*; BR776*: SMC3 WT*; BR777: *SMC3 L1126V*). (H) Western blot analysis of Smc3 acetylation comparing Smc3 L1126V to wild-type (K16297: *eco1-1* BR776*: SMC3 WT*; BR777: *SMC3 L1126V*). Expression of endogenous Smc3 under the control of a galactose-inducible promoter was suppressed on glucose in BR776 and BR777. Cells were grown at 37°C prior to harvesting in order to inactivate Eco1.

Remarkably, the Smc1 L1129V and D1164E mutants did not evidently affect cohesin’s distribution along chromosomes (Figure 4A). While the overall distribution of cohesin remained very similar to wild-type, the amount was reduced by about 30%. At centromeric regions, the decrease was approximately 40% (Figures 4B and S4A), while along arms the decrease was roughly 20% (Figures 4C and S4B). Our results indicate that robust hydrolysis is actually not required to obtain wild-type-like cohesin distribution patterns along chromosomes. This result is in correspondence with our finding that the Smc1 L1129V and D1164E mutants confer good cohesion, as determined by GFP dot assays (Figure 2C), and support viability in the absence of Eco1 (Figures 1C and 2B).

### DNA Release Is Controlled by One of Cohesin’s ATPase Sites

**Figure 5:**
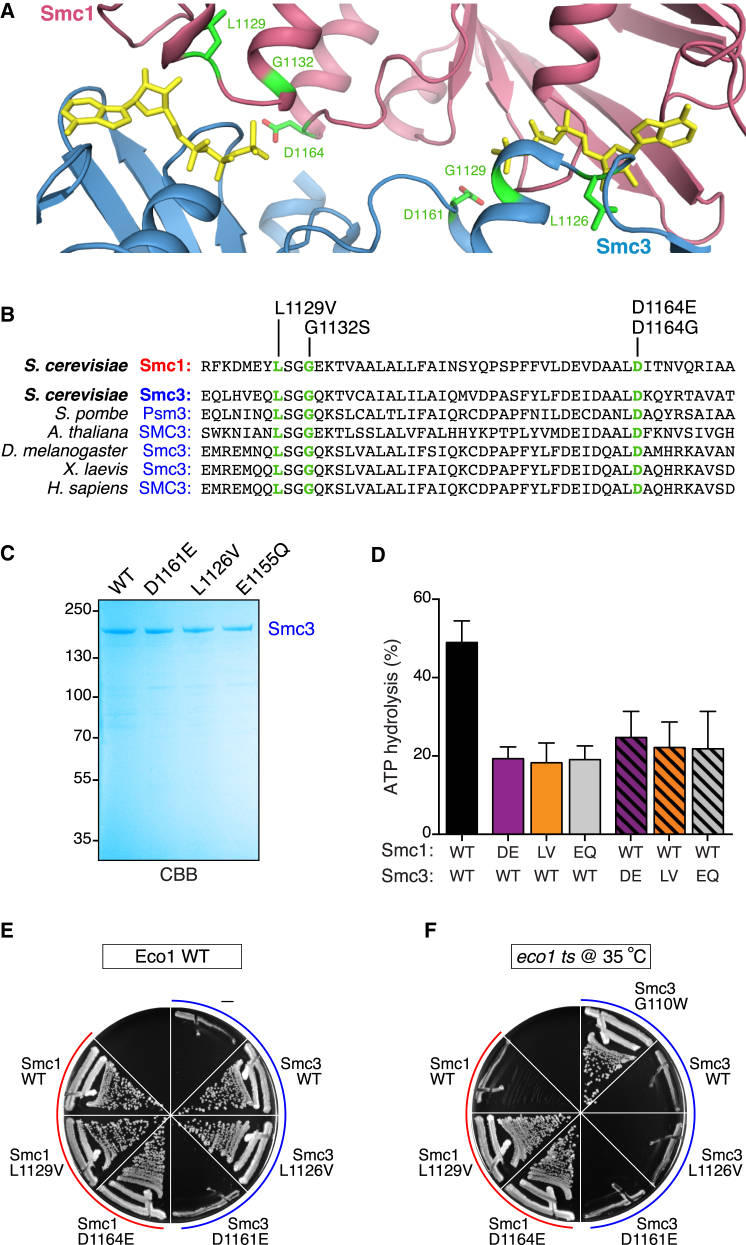
DNA Release Is Controlled by One of Cohesin’s ATPase Sites (A) Model of the Smc1 and Smc3 ATPase heterodimer displaying structural symmetry between these two proteins. The model was assembled based on the crystal structures of Smc1 (red, PDB: 1W1W) and Smc3 (blue, PDB: 4UX3). The mutated Smc1 amino acids and the analogous Smc3 amino acids are depicted in green. (B) The mutated amino acids in Smc1 are conserved in Smc3 from yeast to humans. The amino acid numbers correspond to the *S. cerevisiae* Smc1 protein. (C) SDS-PAGE, Coomassie brilliant blue-stained (CBB) gel depicting a representative prep of either wild-type or mutant Smc3. (D) ATPase assays (as performed in Figure 3B) using either wild-type or the indicated mutant cohesin subunits. The graph depicts ATP hydrolysis after 60 min incubation with [γ-^32^P]-ATP. Error bars show SD of five experiments for the Smc1 mutants and of two experiments for the Smc3 mutants. (E) Smc3 ATPase mutant L1126V does support viability, while Smc3 D1164E does not. Strains harboring wild-type Smc3 under the control of a galactose-inducible promoter, and an ectopic copy of either wild-type or mutant Smc3 were plated on glucose plates at 30°C (streaks left, top to bottom: K699: wild-type; BR420: *SMC1 L1129V*; BR422: *SMC1 D1164E*; streaks right, top to bottom: BR712: *pGAL1-10-SMC3*; BR769: *pGAL1-10-SMC3*, *SMC3 wild-type*; BR770: *pGAL1-10-SMC3*, *SMC3 L1126V*; BR772: *pGAL1-10-SMC3*, *SMC3 D1161E*). See also Figure S5. (F) Smc3 ATPase mutants L1126V and D1161E do not bypass the need for Eco1. Strains harboring a temperature-sensitive *eco1-1* allele, wild-type Smc3 under the control of a galactose-inducible promoter, and an ectopic copy of either wild-type or mutant Smc3 were plated on glucose plates at the non-permissive temperature. Smc3 G110W was used as a positive control (streaks left, top to bottom: K16297: *eco1-1*; BR348: *eco1-1*, *SMC1 L1129V*; BR355: *eco1-1*, *SMC1 D1164E*; streaks right, top to bottom: BR788: *eco1-1*, *pGAL1-10-SMC3*, *SMC3 G110W*; BR787: *eco1-1*, *pGAL1-10-SMC3*, *SMC3 wild-type*; BR774: *eco1-1*, *pGAL1-10-SMC3*, *SMC3 L1126V*; BR775: *eco1-1*, *pGAL1-10-SMC3*, *SMC3 D1161E*).

The ATPase domains of Smc1 and Smc3 are structurally very similar. Together, Smc1 and Smc3 sandwich two ATPs between the respective Signature motif and D-loop of one subunit and the Walker A and Walker B motifs of the other (Figure 5A). However, there is also a certain degree of asymmetry between Smc1 and Smc3’s ATPase domains. For example, only Smc3 is acetylated by Eco1, and structural work shows that Smc3 and Smc1 have different binding modes to the respective N- and C- termini of Scc1 (Gligoris et al., 2014, Haering et al., 2004, Huis in ’t Veld et al., 2014). How and whether this asymmetry is related to ATPase activity is unknown.

Interestingly, the amino acids that we find mutated in the Signature motif and D-loop of Smc1 are also conserved through Smc3 (Figure 5B). We therefore tested whether introducing the analogous mutations into Smc3 yields the same phenotype as the Smc1 mutants. We expressed the recombinant Smc3 mutants (Figure 5C) and tested the effect of these mutations by ATPase assays. We found that the analogous Smc3 mutations L1126V and D1161E significantly reduced cohesin’s ATPase activity in vitro. The effect of these mutations was roughly similar to the Smc3 E1155Q Walker B mutation, but the effect was no greater than the Smc1 L1129V and D1164E mutations (Figure 5D).

We then tested the effect of the Smc3 mutants on the viability of yeast. We used strains that harbored wild-type Smc3 under the control of a galactose-inducible promoter and expressed an ectopic tagless copy of either wild-type or mutant Smc3. Wild-type Smc3 efficiently complemented Smc3 depletion on glucose, but the Smc3 L1126V and D1161E mutants displayed differential effects. Smc3 L1126V supported viability just as well as wild-type Smc3, but Smc3 D1161E caused lethality (Figure 5E).

Next, we studied the cellular localization of PK-tagged Smc3 mutants and found that while Smc3 wild-type and the L1126V mutant were clearly nuclear, the Smc3 D1161E mutant failed to accumulate in the nucleus, and remained largely cytoplasmic (Figure S5A). Smc3 D1161 is predicted to be in close proximity of the Scc1 C-terminal binding interface (Figure S5C). We therefore performed coIP experiments and found that the D1161E mutant was defective in binding to Scc1 (Figure S5E). This suggests that Scc1 C-terminal binding is not only dependent on Smc1, but also on Smc3. Whether this defect in Scc1 binding is a cause or a consequence of the mislocalization is currently unknown. We should note that a number of other mutations in the ATPase domain have been described to prevent nuclear localization (Hu et al., 2011, Beckouët et al., 2016). Due to its lack of nuclear localization, we excluded the Smc3 D1161E mutant from our further analyses.

We then performed calibrated ChIP-seq on Scc1-PK in yeast that expressed an ectopic tagless copy of either wild-type Smc3 or Smc3 L1126V and had the expression of endogenous Smc3 switched off on glucose. Interestingly, the Smc3 L1126V mutation yielded an overall distribution along chromosomes that was very similar to wild-type, but the amount of cohesin at DNA was reduced by roughly 30% (Figures 4E, 4F, S4C, and S4D). We should note that this effect is very much like what we observe for the analogous Smc1 L1129V mutation. Apparently, the two ATPase sites have a similar contribution to the abundance and distribution of cohesin at DNA. We also assessed the effect of the Smc3 L1126V mutation on Smc3 acetylation. This yielded a significant reduction in acetylation relative to wild-type (Figure 4H). This effect was similar to the ATPase mutations in Smc1 (Figure 2A), indicating that the two ATPase sites also have similar contributions to Smc3 acetylation.

As the Smc3 L1126V mutant does support viability, this allowed us to test whether this mutant bypasses the need for Eco1, like the Smc1 mutants L1129V and D1164E. We included Smc3 G110W as a positive control. This latter mutant partially mimics Smc3 acetylation and therefore allows viability without Eco1 (Rowland et al., 2009). Whereas the Smc3 G110W mutant efficiently allowed viability of a temperature-sensitive *eco1-1* strain at the non-permissive temperature on glucose plates, the Smc3 L1126V and D1161E mutants did not (Figure 5F). The absence of a rescue by the Smc3 D1161E mutant is non-informative, as this mutant is not nuclear. The fact that the Smc3 L1126V mutant does not bypass the need for Eco1, however, is an important finding, as this indicates that there is a functional asymmetry within the very heart of cohesin’s ATPase machinery.

Our observation that only Smc1 L1129V, but not Smc3 L1126V, bypasses the need for Eco1 would indicate that the DNA release reaction is only affected by former mutation, but not the latter. Interestingly, the accompanying paper from the Nasmyth laboratory (Beckouët et al., 2016) confirms this finding using an assay that measures the opening of cohesin’s DNA exit gate. Importantly, Smc1 L1129V but not Smc3 L1126V blocked dissociation of this Smc3/Scc1 interface.

### Cohesin’s DNA Release Mechanism Is Conserved from Yeast to Humans

**Figure 6:**
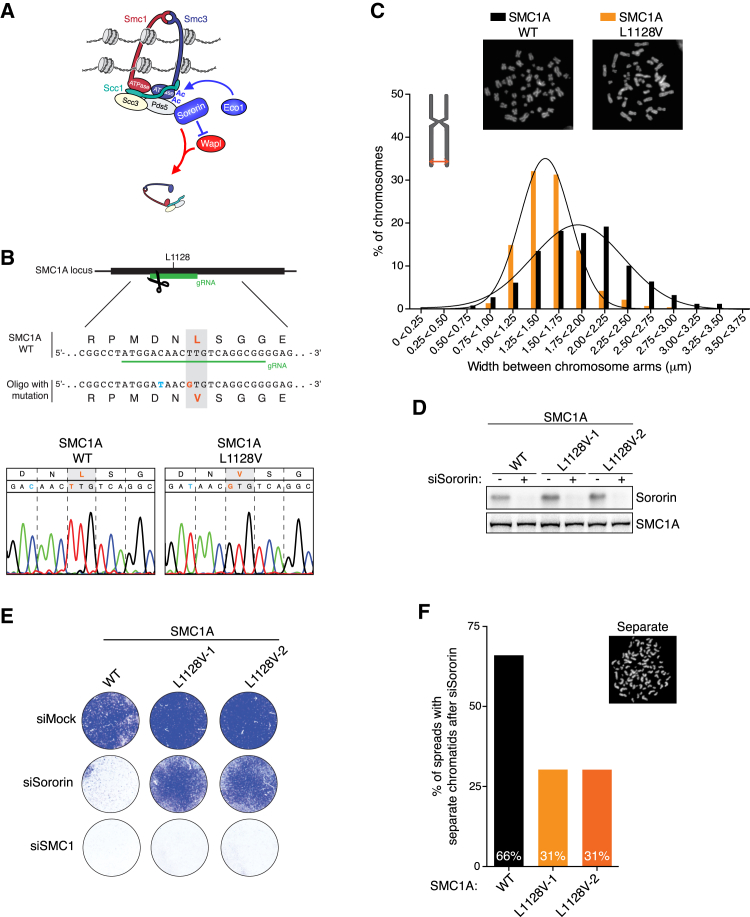
Cohesin’s DNA Release Mechanism Is Conserved from Yeast to Humans (A) In human cells, Sororin is recruited to acetylated cohesin complexes and protects cohesin against Wapl-mediated removal activity. (B) Schematic depiction of CRISPR/Cas9-mediated genome editing, targeting *SMC1A* in human *p53*^−/−^ HCT116 cells. *SMC1A* was cleaved close to the Signature motif, and homology-directed repair was induced by adding a 90 nt donor-oligo harboring the L1128V (T>G) mutation. To prevent re-cleavage of the edited DNA, an additional (silent) mutation (D1127D [C>T]) was introduced within the gRNA recognition site. Sanger sequencing chromatogram of *SMC1A* in wild-type (left) and L1128V mutant (right) cells. (C) Chromosome arms of SMC1A L1128V cells are in closer proximity to each other than in wild-type cells. Cells were treated for one hour with nocodazole prior to harvesting. The distance between sister chromatids was measured for the five largest chromosomes of each spread (as depicted in the model). At least 125 spreads per genotype were analyzed. Images show representative chromosome spreads. (D) Sororin is depleted equally well in control and SMC1A L1128V cells. Total lysate was taken 48 hr after siRNA treatment and analyzed by western blot. (E) SMC1A L1128V mutant cells bypass the need for Sororin. Cells were seeded at equal densities and transfected with siRNAs targeting either Sororin or SMC1A. After 5 days the cells were fixed and stained with Crystal Violet. (F) SMC1A L1128V mutant cells partially rescue the cohesion defects observed upon Sororin depletion. Experiment as in (E). Cells were harvested 2 days post-transfection after 1 hr nocodazole treatment. At least 90 chromosome spreads per genotype were scored.

The amino acids in Smc1’s ATPase domain that we find are key to cohesin’s release from DNA interestingly are conserved through all eukaryotes analyzed, from yeast to humans (Figure 1D). Remarkably though, cohesin’s release is regulated quite differently in humans compared to yeast. A striking example is that in early mitosis of human cells, cohesin is released from chromosome arms in a Wapl-dependent manner, leading to the separation of chromosome arms (Gandhi et al., 2006, Kueng et al., 2006). In budding yeast, however, this “prophase pathway” cohesin removal does not exist, and all cohesin rings are cleaved by Separase at anaphase onset. Also, cohesin’s protection against Wapl is very different. In human cells, Smc3 acetylation allows the recruitment of Sororin, which in turn renders cohesin rings resistant to Wapl (Lafont et al., 2010, Nishiyama et al., 2010) (Figure 6A). Yeast, however, have no Sororin.

We reasoned that even though cohesin release is regulated differently in human cells compared to yeast, the Wapl-dependent cohesin release reaction might nevertheless be fundamentally the same through all eukaryotes. We therefore mutated the endogenous *SMC1A* allele in human cells using CRISPR/Cas9 technology (Figure 6B). Conveniently, *SMC1A* is located on the X chromosome, so we chose the male HCT116 cell line as we only needed to mutate a single allele in these cells. We then made the targeted SMC1A L1128V mutation, which is homologous to the yeast Smc1 L1129V.

Removal of cohesin from chromosome arms is particularly clear in cells artificially arrested in prometaphase with spindle poisons. We therefore arrested control and SMC1A L1128V mutant cells in the spindle poison nocodazole and analyzed chromosome morphology by chromosome spreads. Control cells clearly displayed the classical X shape of human chromosomes, with their fully separated chromosome arms. SMC1A L1128V mutant cells, however, rarely displayed fully separate chromosome arms (Figure 6C). We then systematically measured the distance between the chromosome arms of control and SMC1A L1128V chromosomes. The distance between sister chromatids indeed was smaller in SMC1A L1128V cells than in control cells. This result indicates that the prophase pathway in human cells to a large degree is dependent on the same key amino acid as is cohesin release in budding yeast.

Sororin protects cohesin rings against Wapl from S phase till mitosis. As such, Sororin is essential for viability and cohesion in human cells. Considering that the SMC1A L1128V mutation apparently blocks Wapl-dependent cohesin removal in prophase, we reasoned that this mutation might also bypass the need for Sororin. We therefore knocked down Sororin with siRNAs in control and SMC1A L1128V HCT116 cells and scored for outgrowth in a colony formation assay. As expected, in control cells, Sororin depletion resulted in cell death (Figure 6E). Importantly, the SMC1A L1128V cells continued to propagate despite the equally efficient Sororin knockdown (Figures 6D and 6F).

In parallel, we performed chromosome spreads for these cells. Correspondingly, the SMC1A L1128V mutation significantly reduced the amount of cells with completely separated sister chromatids. While in control cells Sororin depletion resulted in 66% spreads with separated sisters, this number was reduced to 31% in SMC1A L1128V cells (Figure 6F). We obtained virtually identical results using two completely independent SMC1A L1128V cell clones (Smc1A L1128V-1 and Smc1A L1128V-2). Apparently, the SMC1A L1128V mutation does indeed partially bypass the need for Sororin in human cells. Together, these results show that the fundamental basics of the cohesin removal reaction are conserved from yeast to humans.

## Discussion

### Locking Together the Sister DNAs

**Figure 7:**
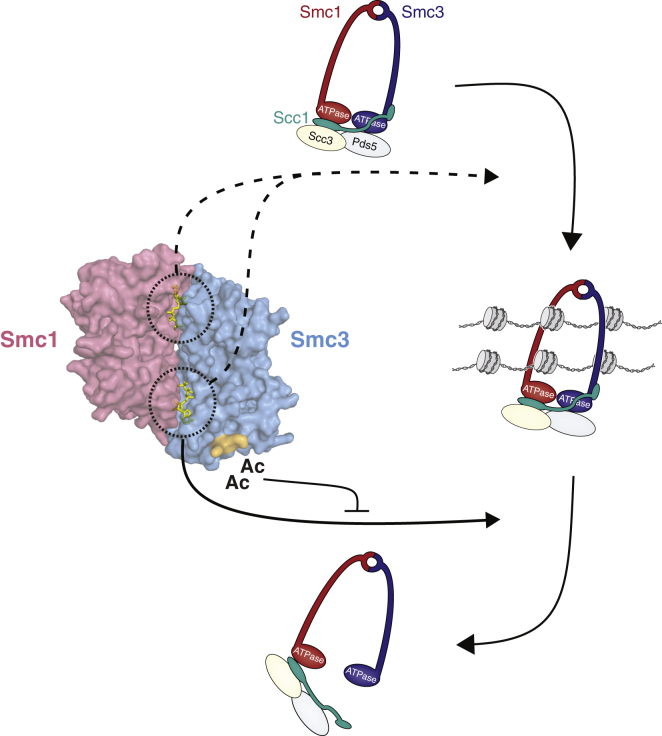
A Model for Asymmetric ATPase-Driven DNA Release by Cohesin DNA release is dependent on an activity associated with one of cohesin’s ATPase sites. We suggest that this activity entails a specific type of ATPase head engagement that causes the dissociation of N-Scc1 from Smc3’s coiled coil and that this engagement is also required for efficient ATP hydrolysis, which allows the passage of DNA out of the cohesin ring. Acetylation of Smc3 at K112 and K113 nearby this specific ATPase site blocks this release activity and thereby locks cohesin around the sister DNAs. Both ATPase sites appear to control DNA entrapment.

We here provide key insight into the cellular mechanism that must be kept in check to allow cohesin to stably hold together the sister chromatids. We find that cohesin’s release from DNA involves a highly conserved asymmetric activity associated with one of cohesin’s ATPase sites. The cohesin removal process in turn is counteracted by the acetylation of two conserved lysines on the outer surface of Smc3 by Eco1. These lysines are in fact located very close to this same ATPase site (Figure 7). Considering that mutants affecting specifically this site bypass the need for Eco1, this allows for the model that Smc3 acetylation locks together the sister chromatids by counteracting an activity associated with this site (Figure 7). We also show that making a homologous SMC1A mutation in human cells bypasses the need for Sororin. This factor is recruited to acetylated cohesin rings in animal cells and is important for the protection against Wapl. Sororin may therefore act to lock cohesin rings around the sister chromatids by preventing this activity. These results also show that the essence of the Wapl-mediated cohesin release mechanism is conserved from yeast to humans.

We identify Smc1 ATPase mutants that are impaired in ATP hydrolysis but that yield viable yeast, good cohesion, and stable DNA association. These mutants are distributed along chromosomes in a pattern that is very similar to wild-type. The key difference compared to wild-type, however, is that these ATPase mutants stabilize cohesin on chromatin and bypass the need for the cohesin protectors Eco1 and Sororin. This result is in stark contrast to previously described Walker B mutants of Smc1 and Smc3. These mutations are lethal to yeast, yield no cohesion or stable DNA binding, and these mutants localize solely to cohesin loading sites on DNA. This difference is remarkable, as the Smc1 L1129V and D1164E mutants are at least as hydrolysis deficient as the Walker B mutants in our ATPase assays.

One possible explanation is that the biological phenotype of the Walker B mutants may not be the consequence of the hydrolysis deficiency, but rather of an unknown additional defect of these mutants. The nature of this defect is currently unknown, but it could, for example, be related to signaling within the complex. It is well possible that these key Walker B amino acids are also involved in relaying the hydrolysis signal to allow the formation of cohesive cohesin complexes. The generally accepted model that ATPase activity is essential for DNA entrapment by cohesin is not purely based on these Walker B mutants though. Recent in vitro work, using non-hydrolyzable ATP, also shows that hydrolysis is required for the entrapment of DNA (Murayama and Uhlmann, 2014). We should note that we are merely inhibiting, but not completely abrogating, ATPase activity with our ATPase mutants. The remaining ATPase activity therefore is likely to be sufficient to allow DNA entrapment.

### Opening the Cohesin Ring

Genetic screens in yeast have been very valuable for the identification of the key regulators of cohesin’s release from DNA. These screens have led to the pinpointing of Eco1’s acetylation targets on Smc3’s ATPase domain, to the finding that this protects cohesin against Wapl-mediated DNA release, and to the mapping of regulatory domains within cohesin subunits (Guacci et al., 2015, Rolef Ben-Shahar et al., 2008, Rowland et al., 2009, Sutani et al., 2009). We designed our current genetic screen such that we could identify mutations in genes that had not hitherto been implicated in cohesin release. This has led us to the identification of mutations within the heart of Smc1’s ATPase domain. Earlier studies have suggested that cohesin’s DNA release may involve ATPase activity (Chatterjee et al., 2013, Guacci et al., 2015, Heidinger-Pauli et al., 2010, Ouyang et al., 2013, Rowland et al., 2009, Unal et al., 2008, Zhang et al., 2008), but direct evidence of this was lacking. We now present Smc1 mutants that are impaired in ATPase activity and indeed cannot release DNA. One of these mutants (Smc1 D1164E) was recently also described in a related study (Çamdere et al., 2015).

A key step in DNA release is the opening up of cohesin’s DNA exit gate, which entails the dissociation of the N terminus of Scc1 (N-Scc1) from the coiled coil of Smc3 located just above the ATPase domain. This raises important questions regarding the chain of events that ultimately leads to the release of DNA from cohesin rings. Notably, neither deletion of N-Scc1 nor mutation of the residues important for its binding to Smc3 has any appreciable effect on hydrolysis (Arumugam et al., 2006, Huis in ’t Veld et al., 2014). Interestingly, the accompanying paper by the Nasmyth laboratory (Beckouët et al., 2016) tested directly whether the Smc1 L1129V and Smc3 L1126V ATPase mutations affected N-Scc1’s association with Smc3. Importantly, Smc1 L1129V, but not Smc3 L1126V, prevents dissociation of N-Scc1 from Smc3. These results raise the possibility that ATP hydrolysis driven by this ATPase site drives opening of cohesin’s DNA exit gate.

Surprisingly, however, an Smc3 E1155Q mutation does not prevent N-Scc1 dissociation. Thus, two different mutations (Smc1 L1129V and Smc3 E1155Q) that both affect the same ATPase site, and also both reduce ATP hydrolysis, have very different effects on N-Scc1 release. One possible explanation is that Smc1 L1129V affects the ATPase cycle at an earlier step than Smc3 E1155Q. As Smc1 L1129V complexes can engage their ATPase heads, but are impaired in hydrolysis, this suggest that there is a previously unreported but apparently very important step between ATP-dependent head engagement and ATP hydrolysis. What this step entails in molecular terms remains unknown, but this is likely to involve a conformational change within the head domains that results in an optimal orientation of the ATPase heads for hydrolysis. In this particular scenario, this conformational change would also serve another crucial role, namely the dissociation of N-Scc1 from Smc3’s coiled coil.

We should note that even if hydrolysis itself does not directly drive N-Scc1 dissociation, ATP hydrolysis is still likely to be a key event for DNA release from cohesin rings. If cohesin’s ATPase heads indeed engage prior to DNA release, these heads would presumably need to separate to allow the passage of DNA through this interface out of cohesin’s lumen. ATP hydrolysis would be the perfect way to achieve this separation and subsequent DNA release.

Previous work has shown that cohesin-mediated ATPase activity is required for Smc3 acetylation, which in turn is key to locking cohesin rings on the DNA, and that Smc3 acetylation does not affect cohesin’s ATPase activity in vitro (Ladurner et al., 2014). This finding appears contradictory to the model that ATPase activity acts both upstream and downstream of DNA entrapment and that Smc3 acetylation prevents the second hydrolysis step. In this setting, we should note that Eco1 appears to only acetylate cohesive cohesin complexes, which by definition only takes place in the context of DNA. We therefore suggest that Smc3 acetylation may only act to inhibit ATPase activity of cohesin complexes that have co-entrapped the sister DNAs (Figure 7). How Eco1 knows which cohesin complexes to acetylate remains one of the main open questions in the field.

### An Asymmetric Activity within Cohesin’s ATPase Machinery

Our finding that the analogous mutants Smc1 L1129V and Smc3 L1126V both yield the same 30% reduction in cohesin’s abundance on chromatin at first sight may be considered to indicate that each of these mutations affects cohesin’s loading onto DNA to the same degree. We should, however, realize that the total abundance of cohesin at DNA is the balance of an on-rate in DNA loading and an off-rate through DNA release. As the Smc1 L1129V mutation affects cohesin’s off-rate, and the Smc3 L1126V mutation as far as we can tell does not, this may indicate that the on-rates of these different mutants are in fact very different. If anything, this would suggest that the Smc1 L1129V mutation reduces cohesin’s on-rate to a stronger degree than Smc3 L1126V and that this decrease is masked by an effect on cohesin’s off-rate. In that case, this particular ATPase site might actually be the main driver of both entrapment and release.

If so, one would expect that this ATPase site is more important for hydrolysis than the other. This, however, does not appear to be the case for the yeast cohesin complex, as inactivation of each ATPase site merely reduces hydrolysis while this is further reduced upon the inactivation of both sites (Figure 3B) (Arumugam et al., 2006)). Remarkably, this may be different for the human cohesin complex, as mutation of one site completely abrogates ATPase activity, while the other site is less important (Ladurner et al., 2014). Interestingly, this key ATPase site in humans appears to be the same site that we suggest could be the main driver of both entrapment and release. This is the site that harbors the Signature motif and D-loop of Smc1 and the Walker A and Walker B motifs of Smc3. This evidently is something that needs further investigation.

Tight control of DNA entrapment and release by the cohesin complex is critical for faithful chromosome segregation in mitosis but may be equally important for DNA repair and transcriptional regulation. Cohesin ensures the proximity of an undamaged sister DNA template to allow high-fidelity repair through homologous recombination, and it is also essential for the formation or maintenance of DNA loops that control gene expression. Cohesin could in essence be viewed as a “chromatin transporter” that transports DNA in and out of its lumen. The transporter analogy stretches further, as cohesin’s ATPase domain is very similar to that of ABC-like transporters (Haarhuis et al., 2014). In this setting it is worth pointing out that ABC-like transporters can display differential roles for their two associated ATPs (Procko et al., 2009). It is therefore likely that an asymmetric division of tasks reflects a universal theme among ABC-like ATPases.

Cohesin is the best understood of three structurally similar Smc protein complexes. The condensin complex (with an Smc2/Smc4 heterodimer at its basis) and the Smc5/Smc6 complex are important for chromosome condensation and DNA repair, respectively (Nasmyth and Haering, 2009). Notably, the amino acids that we pinpoint as being key to cohesin’s removal from DNA are conserved through these three complexes. This raises crucial questions about the potential functional conservation of the cycle of chromatin entrapment and release throughout this important family of protein complexes.

## Experimental Procedures

### Yeast Genetics and In Vivo Characterization

All yeast strains are derivatives of W303 (K699) and were grown on YEPD plates at 30°C unless otherwise specified. No more that one *eco1-1* suppressor was isolated from each of 500 independent parental clones at 35°C. Suppressors were identified by complementation group analysis, followed by deep sequencing of the genomic DNA from suppressors that had no mutations in *WAPL*, *SMC3*, *SCC3*, or *PDS5*. Cohesion was scored by GFP dot assays, cohesin’s turnover on DNA by Scc1-FRB-GFP anchor away assays, and cohesin’s abundance on DNA by calibrated ChIP-seq analyses.

### Biochemistry

DNA sequences encoding the *S. cerevisiae* Smc1, Smc3, and Scc1 C-terminal part were amplified by PCR and cloned into the Bac-to-Bac pFastBac NKI-LIC expression vectors. All proteins are N-terminally tagged and were expressed in Sf9 insect cells. Co-expressed Smc1 and Scc1-C were purified using nickel affinity purification, followed by a Strep-II tag purification step. Smc3 was purified using Strep-II tag purification, followed by a size-exclusion chromatography step. Purified proteins were co-incubated prior to scoring for ATPase head engagement by scanning force microscopy (SFM), measuring ATP hydrolysis using [γ-^32^P]-ATP and thin-layer chromatography, and fluorescently labeling the cohesin subunits for microscale thermophoresis assays (MST). Thermophoresis was measured to assess ATP or ADP binding.

### Experiments in Human Cells

HCT116 *p53*^−/−^ cells were genome-edited using CRISPR/Cas9 technology. Cohesion was scored by chromosome spreads. For depletion of Sororin, cells were transfected with siRNAs and subsequently analyzed by colony formation assays, chromosome spreads, or western blot analysis.

See Supplemental Experimental Procedures for further details.

## Accession Numbers

The GEO accession number for the calibrated ChIP-seq data reported in this paper is GEO: GSE76891.

## Supplemental Information

Document S1. Figures S1–S5, Tables S1 and S2, and Supplemental Experimental Procedures

Document S2. Article plus Supplemental Information

## References

[bib1] Arumugam P., Gruber S., Tanaka K., Haering C.H., Mechtler K., Nasmyth K. (2003). ATP hydrolysis is required for cohesin’s association with chromosomes. Curr. Biol..

[bib2] Arumugam P., Nishino T., Haering C.H., Gruber S., Nasmyth K. (2006). Cohesin’s ATPase activity is stimulated by the C-terminal Winged-Helix domain of its kleisin subunit. Curr. Biol..

[bib3] Beckouët F., Srinivasan M., Roig M.B., Chan K.L., Scheinost J.C., Batty P., Petela N., Gligoris T., Smith A.C., Strmecki L. (2016). Releasing activity disengages cohesin’s Smc3/Scc1 interface in a process blocked by acetylation. Mol. Cell.

[bib4] Buheitel J., Stemmann O. (2013). Prophase pathway-dependent removal of cohesin from human chromosomes requires opening of the Smc3-Scc1 gate. EMBO J..

[bib5] Çamdere G., Guacci V., Stricklin J., Koshland D. (2015). The ATPases of cohesin interface with regulators to modulate cohesin-mediated DNA tethering. eLife.

[bib6] Chan K.L., Roig M.B., Hu B., Beckouët F., Metson J., Nasmyth K. (2012). Cohesin’s DNA exit gate is distinct from its entrance gate and is regulated by acetylation. Cell.

[bib7] Chatterjee A., Zakian S., Hu X.-W., Singleton M.R. (2013). Structural insights into the regulation of cohesion establishment by Wpl1. EMBO J..

[bib8] Ciosk R., Shirayama M., Shevchenko A., Tanaka T., Tóth A., Shevchenko A., Nasmyth K. (2000). Cohesin’s binding to chromosomes depends on a separate complex consisting of Scc2 and Scc4 proteins. Mol. Cell.

[bib9] Eichinger C.S., Kurze A., Oliveira R.A., Nasmyth K. (2013). Disengaging the Smc3/kleisin interface releases cohesin from Drosophila chromosomes during interphase and mitosis. EMBO J..

[bib10] Gandhi R., Gillespie P.J., Hirano T. (2006). Human Wapl is a cohesin-binding protein that promotes sister-chromatid resolution in mitotic prophase. Curr. Biol..

[bib11] Gerlich D., Koch B., Dupeux F., Peters J.-M., Ellenberg J. (2006). Live-cell imaging reveals a stable cohesin-chromatin interaction after but not before DNA replication. Curr. Biol..

[bib12] Gligoris T.G., Scheinost J.C., Bürmann F., Petela N., Chan K.L., Uluocak P., Beckouët F., Gruber S., Nasmyth K., Löwe J. (2014). Closing the cohesin ring: structure and function of its Smc3-kleisin interface. Science.

[bib13] Gruber S., Haering C.H., Nasmyth K. (2003). Chromosomal cohesin forms a ring. Cell.

[bib14] Guacci V., Stricklin J., Bloom M.S., Guō X., Bhatter M., Koshland D. (2015). A novel mechanism for the establishment of sister chromatid cohesion by the ECO1 acetyltransferase. Mol. Biol. Cell.

[bib15] Haarhuis J.H.I., Elbatsh A.M.O., Rowland B.D. (2014). Cohesin and its regulation: on the logic of X-shaped chromosomes. Dev. Cell.

[bib16] Haering C.H., Löwe J., Hochwagen A., Nasmyth K. (2002). Molecular architecture of SMC proteins and the yeast cohesin complex. Mol. Cell.

[bib17] Haering C.H., Schoffnegger D., Nishino T., Helmhart W., Nasmyth K., Löwe J. (2004). Structure and stability of cohesin’s Smc1-kleisin interaction. Mol. Cell.

[bib18] Haering C.H., Farcas A.-M., Arumugam P., Metson J., Nasmyth K. (2008). The cohesin ring concatenates sister DNA molecules. Nature.

[bib19] Hara K., Zheng G., Qu Q., Liu H., Ouyang Z., Chen Z., Tomchick D.R., Yu H. (2014). Structure of cohesin subcomplex pinpoints direct shugoshin-Wapl antagonism in centromeric cohesion. Nat. Struct. Mol. Biol..

[bib20] Haruki H., Nishikawa J., Laemmli U.K. (2008). The anchor-away technique: rapid, conditional establishment of yeast mutant phenotypes. Mol. Cell.

[bib21] Heidinger-Pauli J.M., Onn I., Koshland D. (2010). Genetic evidence that the acetylation of the Smc3p subunit of cohesin modulates its ATP-bound state to promote cohesion establishment in Saccharomyces cerevisiae. Genetics.

[bib22] Hu B., Itoh T., Mishra A., Katoh Y., Chan K.L., Upcher W., Godlee C., Roig M.B., Shirahige K., Nasmyth K. (2011). ATP hydrolysis is required for relocating cohesin from sites occupied by its Scc2/4 loading complex. Curr. Biol..

[bib23] Hu B., Petela N., Kurze A., Chan K.L., Chapard C., Nasmyth K. (2015). Biological chromodynamics: a general method for measuring protein occupancy across the genome by calibrating ChIP-seq. Nucleic Acids Res..

[bib24] Huis in ’t Veld P.J., Herzog F., Ladurner R., Davidson I.F., Piric S., Kreidl E., Bhaskara V., Aebersold R., Peters J.-M. (2014). Characterization of a DNA exit gate in the human cohesin ring. Science.

[bib25] Kueng S., Hegemann B., Peters B.H., Lipp J.J., Schleiffer A., Mechtler K., Peters J.-M. (2006). Wapl controls the dynamic association of cohesin with chromatin. Cell.

[bib26] Ladurner R., Bhaskara V., Huis in ’t Veld P.J., Davidson I.F., Kreidl E., Petzold G., Peters J.-M. (2014). Cohesin’s ATPase activity couples cohesin loading onto DNA with Smc3 acetylation. Curr. Biol..

[bib27] Lafont A.L., Song J., Rankin S. (2010). Sororin cooperates with the acetyltransferase Eco2 to ensure DNA replication-dependent sister chromatid cohesion. Proc. Natl. Acad. Sci. USA.

[bib28] Lopez-Serra L., Lengronne A., Borges V., Kelly G., Uhlmann F. (2013). Budding yeast Wapl controls sister chromatid cohesion maintenance and chromosome condensation. Curr. Biol..

[bib29] Michaelis C., Ciosk R., Nasmyth K. (1997). Cohesins: chromosomal proteins that prevent premature separation of sister chromatids. Cell.

[bib30] Murayama Y., Uhlmann F. (2014). Biochemical reconstitution of topological DNA binding by the cohesin ring. Nature.

[bib31] Nasmyth K., Haering C.H. (2009). Cohesin: its roles and mechanisms. Annu. Rev. Genet..

[bib32] Nishiyama T., Ladurner R., Schmitz J., Kreidl E., Schleiffer A., Bhaskara V., Bando M., Shirahige K., Hyman A.A., Mechtler K., Peters J.M. (2010). Sororin mediates sister chromatid cohesion by antagonizing Wapl. Cell.

[bib33] Ouyang Z., Zheng G., Song J., Borek D.M., Otwinowski Z., Brautigam C.A., Tomchick D.R., Rankin S., Yu H. (2013). Structure of the human cohesin inhibitor Wapl. Proc. Natl. Acad. Sci. USA.

[bib34] Peters J.-M., Tedeschi A., Schmitz J. (2008). The cohesin complex and its roles in chromosome biology. Genes Dev..

[bib35] Procko E., O’Mara M.L., Bennett W.F.D., Tieleman D.P., Gaudet R. (2009). The mechanism of ABC transporters: general lessons from structural and functional studies of an antigenic peptide transporter. FASEB J..

[bib36] Rolef Ben-Shahar T., Heeger S., Lehane C., East P., Flynn H., Skehel M., Uhlmann F. (2008). Eco1-dependent cohesin acetylation during establishment of sister chromatid cohesion. Science.

[bib37] Rowland B.D., Roig M.B., Nishino T., Kurze A., Uluocak P., Mishra A., Beckouët F., Underwood P., Metson J., Imre R. (2009). Building sister chromatid cohesion: smc3 acetylation counteracts an antiestablishment activity. Mol. Cell.

[bib38] Shintomi K., Hirano T. (2009). Releasing cohesin from chromosome arms in early mitosis: opposing actions of Wapl-Pds5 and Sgo1. Genes Dev..

[bib39] Sutani T., Kawaguchi T., Kanno R., Itoh T., Shirahige K. (2009). Budding yeast Wpl1(Rad61)-Pds5 complex counteracts sister chromatid cohesion-establishing reaction. Curr. Biol..

[bib40] Unal E., Heidinger-Pauli J.M., Kim W., Guacci V., Onn I., Gygi S.P., Koshland D.E. (2008). A molecular determinant for the establishment of sister chromatid cohesion. Science.

[bib41] Weitzer S., Lehane C., Uhlmann F. (2003). A model for ATP hydrolysis-dependent binding of cohesin to DNA. Curr. Biol..

[bib42] Zhang J., Shi X., Li Y., Kim B.-J., Jia J., Huang Z., Yang T., Fu X., Jung S.Y., Wang Y. (2008). Acetylation of Smc3 by Eco1 is required for S phase sister chromatid cohesion in both human and yeast. Mol. Cell.

