# Supplementary material for: Cohesin Releases DNA through Asymmetric ATPase-Driven Ring Opening
Source: Mol Cell. 2016 Feb 18;61(4):575–88. doi: 10.1016/j.molcel.2016.01.025 (PMC4769319; doi:10.1016/j.molcel.2016.01.025)
Supplement: Document S1. Figures S1–S5, Tables S1 and S2, and Supplemental Experimental Procedures [file mmc1.pdf]

Molecular Cell, Volume *61*

Supplemental Information

Cohesin Releases DNA through Asymmetric ATPase-Driven Ring Opening

Ahmed M.O. Elbatsh, Judith H.I. Haarhuis, Naomi Petela, Christophe Chapard, Alexander Fish, Patrick H. Celie, Magda Stadnik, Dejan Ristic, Claire Wyman, René H. Medema, Kim Nasmyth, and Benjamin D. Rowland

Figure S1

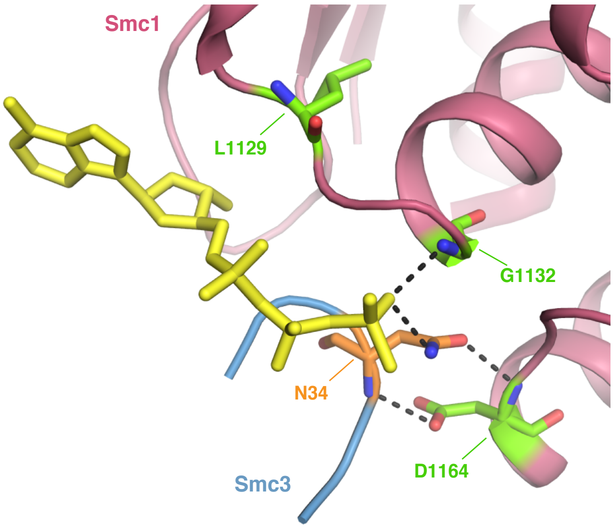

Supplemental Figure 1 - Related to Figure 1
Detailed depiction of interactions between mutated amino acids in Smc1 and ATP
Structure of the ATPase domain of Smc1 (PDB: 1W1W) showing the close proximity of mutated residues G1132 and D1164 to the gamma phosphate of ATP. The mutated Smc1 L1129 is close to ATP's sugar. Dashed lines depict polar bonds. The model depicts Smc3's loop based on the Smc1 crystal structure.

Figure S2

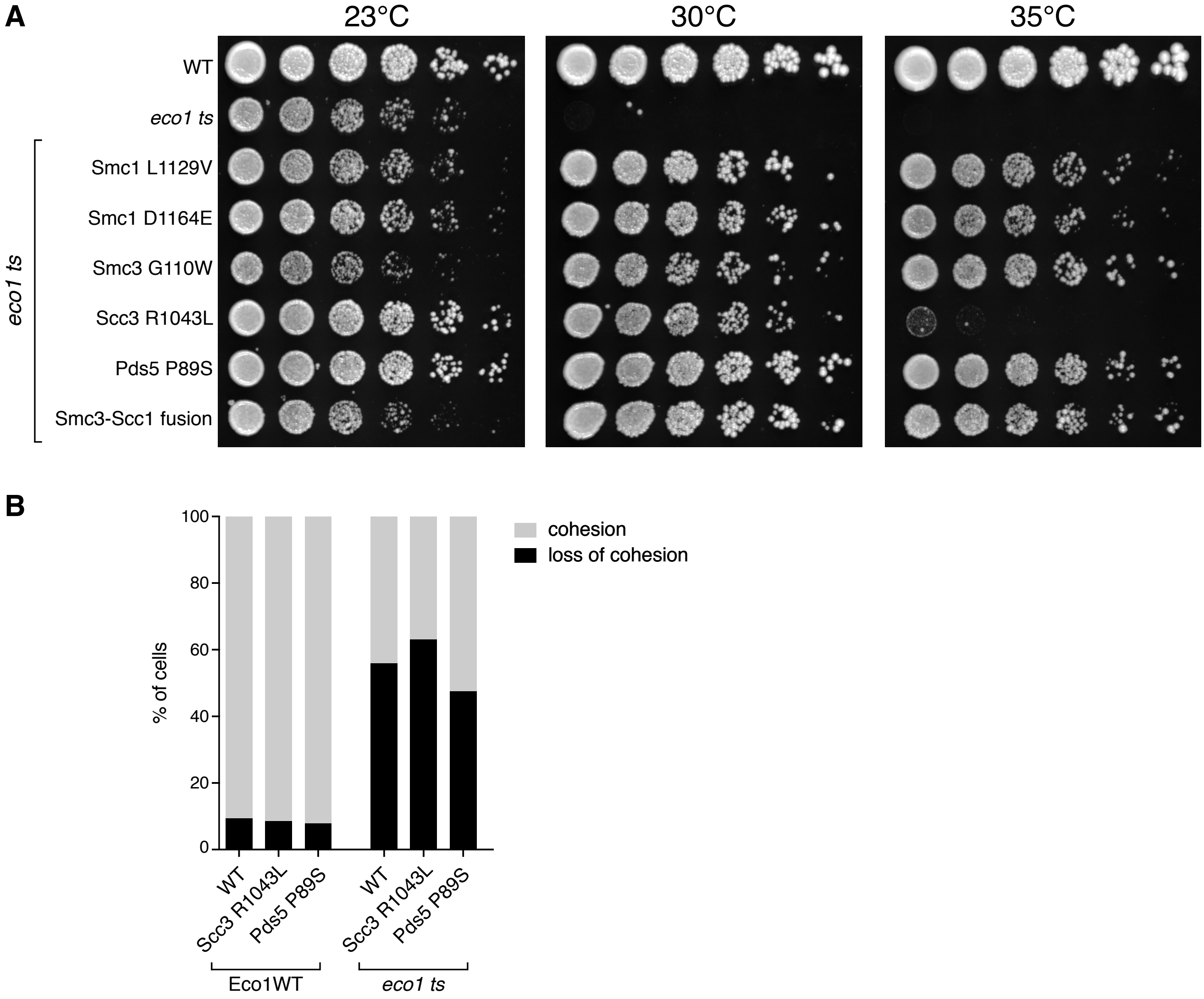

Figure S2 - Related to Figure 1 and 2
Comparison of *eco1-1* suppressors
(A) Mutations in Smc1's ATPase domain rescue the lethality of *eco1 ts* at non-permissive temperatures (K699: *Wild Type*; K16297: *eco1-1;* BR348: *SMC1 L1129V, eco1-1;* BR355: *SMC1 D1164E, eco1-1;* BR783: *SMC3 G110W, eco1-1;* BR784: *SCC3 R1043L, eco1-1;* BR785: *Pds5 P89S, eco1-1;* BR782: *SMC3-SCC1 fusion, eco1-1*). Cells were grown at OD_{600} 0.6, diluted 1:4 and subsequently spotted on YPD plates at the indicated temperatures. Photographs were taken after 72 hours (23°C) or 48 hours (30°C and 35°C).
(B) Percentage of cells with cohesed or separated GFP dots marking the *URA3* locus in wild type (BR455: *ECO1* and BR426: *eco1-1*), Scc3 R1043L (BR778: *ECO1* and BR779: *eco1-1*) and Pds5 P89S (BR780: *ECO1* and BR781: *eco1-1*) yeast. Cells were synchronized in G1 and released at the non-permissive temperature. Cohesion was scored in metaphase-arrested cells. For GFP dot assays with Smc1 ATPase mutants see Figure 2C.

Figure S3

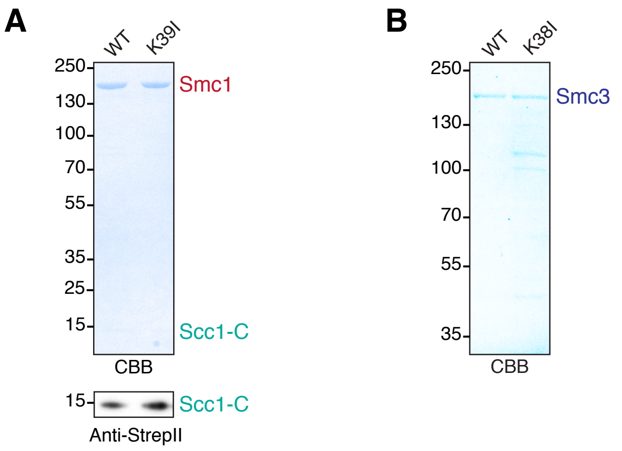

Figure S3 - Related to Figure 3

Expression of recombinant Smc1 K39I and Smc3 K38I mutants used for MST and SFM assays in Figure 3E, F and G

(A) Recombinant expression of cohesin subunits. SDS-PAGE, Coomassie brilliant blue staining (CBB) and western blots of full-length HIS_6-Smc1 (wild type and K39I mutant) and the co-expressed C-terminus of Scc1 (StrepII-Scc1-C).

(B) SDS-PAGE, Coomassie brilliant blue staining of recombinant expression of StrepII-Smc3 (wild type and K38I mutant).

Figure S4

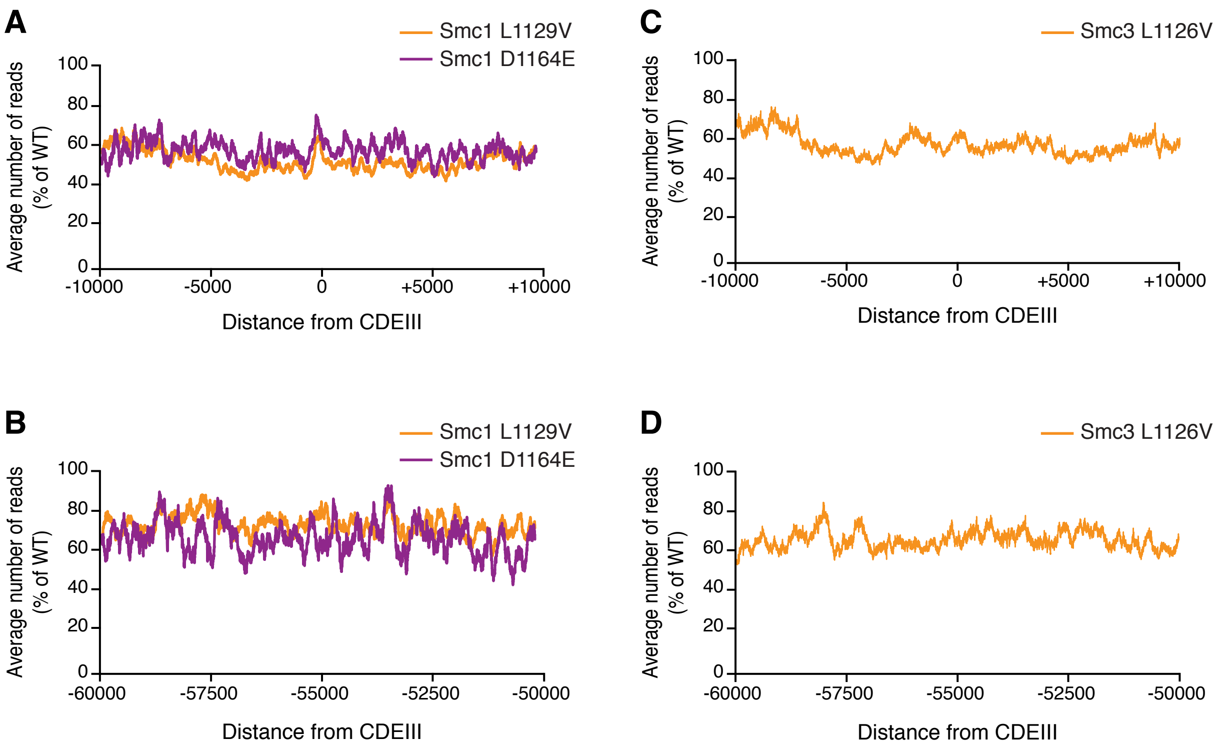

Figure S4 - Related to Figure 4

Smc1 and Smc3 ATPase mutants reduce cohesin's abundance on DNA

(A) Smc1 ATPase mutants show a global decrease of approximately 40% of Scc1-PK binding within centromeric and pericentromeric regions. The plot depicts the data as in (Figure 4B), but displays mutant binding relative to wild type.

(B) Smc1 ATPase mutants show a decrease of approximately 30% of Scc1-PK binding at arm regions. The plot depicts the data as in (Figure 4C), but displays mutant binding relative to wild type.

(C) As in (A) but comparing Smc3 L1126V to Smc3 wild type cells. The Smc3 ATPase mutant shows a similar decrease of approximately 40% of Scc1-PK binding to centromeric and pericentromeric DNA as the corresponding Smc1 ATPase mutant.

(D) As in (B) but comparing Smc3 L1126V to Smc3 wild type cells. The Smc3 ATPase mutant shows a similar decrease of approximately 30% of Scc1-PK binding to the arm regions of chromosomes as the corresponding Smc1 ATPase mutant.

Supplemental Figure 5

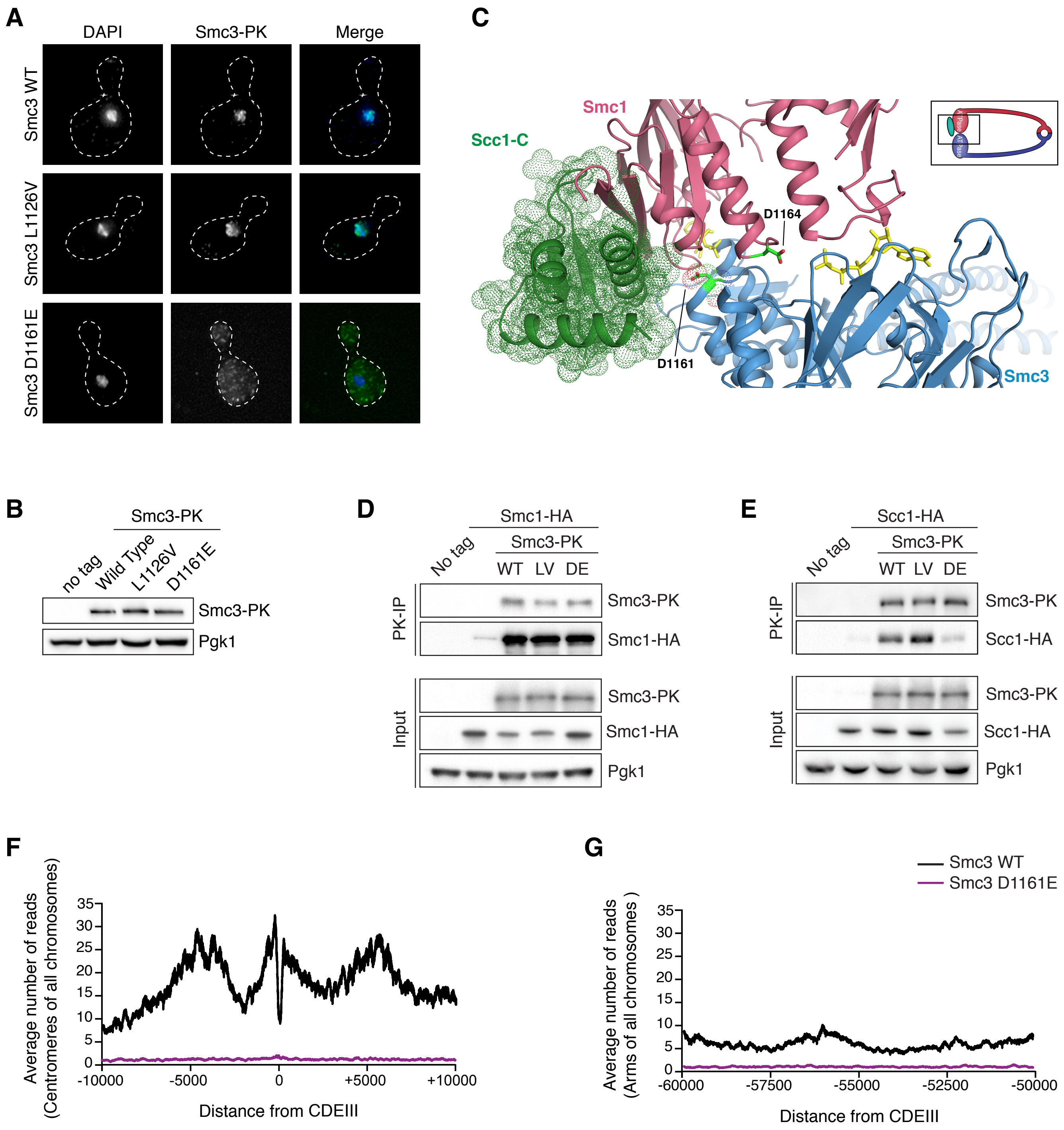

Supplemental Figure 5 - Related to Figure 5
Smc3 D1161E does not enter the nucleus

(A) Smc3 D1161E fails to accumulate in the nucleus, whereas Smc3 WT and L1126V do (BR733: *SMC3-WT-PK*; BR735: *SMC3-L1126V-PK*; BR736: *SMC3-D1161E-PK*). Image brightness was increased for Smc3 D1161E-PK for visualisation purposes.

(B) Expression levels of Smc3 L1126V and Smc3 D1161E mutants are similar compared to wild type Smc3 (K699: *Wild Type No tag*; BR733: *SMC3-WT-PK*; BR735: *SMC3-L1126V-PK*; BR736: *SMC3-D1161E-PK*).

(C) Model depicting the interface of Scc1's C-terminus with the head domains of Smc1 and Smc3. Smc3 D1161E is predicted to be at the Scc1 binding interface. The model was assembled as in Figure 5A.

(D) Smc3 L1126V and D1161E mutants bind to Smc1 as wild type (K699: *Wild Type No tag*; BR786: *SMC1-HA;* BR745: *SMC1-HA, SMC3-WT-PK*; BR753: *SMC1-HA, SMC3-L1126V-PK*; BR761: *SMC1-HA, SMC3-D1161E-PK.*

(E) Smc3 D1161E mutant is unable to form a complex with Scc1, whereas Smc3 L1126V shows similar binding as wild type Smc3 (K699: *Wild Type No tag*; BR742: *SCC1-HA*; BR746: *SCC1-HA, SMC3-WT-PK*; BR754: *SCC1-HA, SMC3-L1126V-PK*; BR762: *SCC1-HA, SMC3-D1161E-PK*).

(F) Smc3 D1161E-PK does not localise to centromeres. The plot depicts the average distribution of cohesin around the centromere (CDEIII) of all chromosomes (BR733: *SMC3-WT-PK*; BR736: *SMC3-D11161E-PK*). Smc3 WT is the same as used in Figure 4E.

(G) As in (E) but at arm regions. The plot depicts the average distribution of Smc3-PK at arm regions spanning from 60 to 50 kb from the centromere (CDEIII) of all chromosomes. Smc3 WT is the same as used in Figure 4F.

Table S1

Smc subunit	K_D for ATP binding (µM ± SEM)	K_D for ADP binding (µM ± SEM)
Smc1-WT	442 ± 176	1989 ± 878
Smc1-L1129V	667 ± 211	2406 ± 958
Smc1-D1164E	542 ± 188	1716 ± 576
Smc1-E1158Q	567 ± 289	1653 ± 601
Smc1-K39I	not detected	not detected
Smc3-K38I	not detected	not detected

Table S1 - Related to Figure 3
Dissociation constant for Smc1 or Smc3 ATPase mutants

Dissociation constant (K_D) values obtained from MST binding curves of titrated ATP or ADP against fluorescently labelled cohesin subunits as depicted in Figure 3E and F respectively. Wild type or mutants Smc1 were pre-incubated with Scc1-C and wild type or mutant Smc3 prior to labelling.

Table S2

Strain nr.	Genotype
BR348	*MAT**a**, eco1-1, SMC1 L1129V*
BR355	*MAT**a**, eco1-1, SMC1 D1164E*
BR363	*Matα, eco1-1, SMC1 D1164G*
BR420	*MAT**a**, SMC1 L1129V*
BR422	*MAT**a**, SMC1 D1164E*
BR426	*MAT**a**, eco1-1, ura3::3XURA3 TetO112, his3::HIS3tetR-GFP, TRP1::MET3promoter-Cdc20*
BR428	*MAT**a**, eco1-1, SMC1 L1129V, ura3::3XURA3 TetO112, his3::HIS3tetR-GFP, TRP1::MET3promoter-Cdc20*
BR429	*MAT**a**, eco1-1, SMC1 D1164E, ura3::3XURA3 TetO112, his3::HIS3tetR-GFP, TRP1::MET3promoter-Cdc20*
BR431	*MAT**a**, eco1-aid::KAN, RPL13A-FKBP12:TRP1, TOR1-1, fpr1Δ::NatMX, SCC1-FRB-GFP::HIS3, ADH1promoter-OsTIR1myc9::URA3*
BR433	*MAT**a**, eco1-aid::KAN, wpl1Δ::LEU2, RPL13A-FKBP12:TRP1, TOR1-1, fpr1Δ::NatMX, SCC1-FRB-GFP::HIS3, ADH1promoter-OsTIR1myc9::URA3*
BR439	*MAT**a**, RPL13A-FKBP12:TRP1, TOR1-1, fpr1Δ::NatMX, SCC1-FRB-GFP::HIS3, ADH1promoter-OsTIR1myc9::URA3*
BR445	*MAT**a**, eco1-aid::KAN, SMC1 D1164E, RPL13A-FKBP12:TRP1, TOR1-1, fpr1Δ::NatMX, SCC1-FRB-GFP::HIS3, ADH1promoter-OsTIR1myc9::URA3*
BR448	*MAT**a**, eco1-1, SMC1 G1132S*
BR455	*MAT**a**, ECO1 WT-NAT, ura3::3XURA3 TetO112, his3::HIS3tetR-GFP, TRP1::MET3promoter-Cdc20*
BR459	*MAT**a**, SMC1 L1129V, ECO1 WT-NAT, ura3::3XURA3 TetO112, his3::HIS3tetR-GFP, TRP1::MET3promoter-Cdc20*
BR461	*MAT**a**, SMC1 D1164E, ECO1 WT-NAT, ura3::3XURA3 TetO112, his3::HIS3tetR-GFP, TRP1::MET3promoter-Cdc20*
BR463	*Diploid, eco1Δ::KanMX / ECO1 WT, SMC1 L1129V / SMC1 WT-HA3::HIS3*
BR464	*Diploid, eco1Δ::KanMX / ECO1 WT, SMC1 D1164E / SMC1 WT-HA3::HIS3*
BR572	*MAT**a**, eco1-aid::KAN, SMC1 L1129V, RPL13A-FKBP12:TRP1, TOR1-1, fpr1Δ::NatMX, SCC1-FRB-GFP::HIS3, ADH1promoter-OsTIR1myc9::URA3*
BR625	*MAT**a**, SMC1 L1129V, SCC1-PK9::KAN*
BR627	*MAT**a**, SMC1 D1164E, SCC1-PK9::KAN*
BR629	*MAT**a**, SMC1 D1164G, SCC1-PK9::KAN*
BR643	*MAT**a**, SMC1 G1132S, SCC1-PK9::KAN*
BR645	*MAT**a**, SCC1-PK9::KAN*
BR651	*MAT**a**, SMC1 L1129V, WPL1-MYC18::URA3*
BR653	*MAT**a**, SMC1 G1132S, WPL1-MYC18::URA3*
BR655	*MAT**a**, SMC1 D1164E, WPL1-MYC18::URA3*
BR657	*MAT**a**, SMC1 D1164G, WPL1-MYC18::URA3*
BR733	*MAT**a**, trp1::SMC3-WT-PK6::TRP1*
BR735	*MAT**a**, trp1::SMC3-L1126V-PK6::TRP1*
BR736	*MAT**a**, trp1::SMC3-D1161E-PK6::TRP1*
BR742	*MAT**a**, leu2::SCC1-HA3::LEU2*
BR745	*MATα, SMC1-HA6::HIS3, trp1::SMC3-WT-PK6::TRP1*
BR746	*MAT**a**, leu2::SCC1-HA3::LEU2, trp1::SMC3-WT-PK6::TRP1*
BR753	*MATα, SMC1-HA6::HIS3, trp1::SMC3-L1126V-PK6::TRP1*
BR754	*MAT**a**, leu2::SCC1-HA3::LEU2, trp1::SMC3-L1126V-PK6::TRP1*
BR761	*MATα, SMC1-HA6::HIS3, trp1::SMC3-D1161E-PK6::TRP1*
BR762	*MAT**a**, leu2::SCC1-HA3::LEU2, trp1::SMC3-D1161E-PK6::TRP1*
BR769	*MAT**a**, KAN-pGal-10-SMC3, leu2::SMC3 WT (Tagless)::LEU2*
BR770	*MAT**a**, KAN-pGal1-10-SMC3, leu2::SMC3 L1126V (Tagless)::LEU2*
BR772	*MAT**a**, KAN-pGal1-10-SMC3, leu2::SMC3 D1161E (Tagless)::LEU2*
BR774	*MAT**a**, eco1-1, KAN-pGal1-10-SMC3, leu2::SMC3 L1126V (Tagless)::LEU2*
BR775	*MAT**a**, eco1-1, KAN-pGal1-10-SMC3, leu2::SMC3 D1161E (Tagless)::LEU2*
BR776	*MAT**a**, KAN-pGal-10-SMC3, leu2::SMC3 WT (Tagless)::LEU2, SCC1-PK9::KAN*
BR777	*MAT**a**, KAN-pGal-10-SMC3, leu2::SMC3 L1126V (Tagless)::LEU2, SCC1-PK9::KAN*
BR778	*MATα, ECO1 WT-NatMX, SCC3 R1043L, ura3::3XURA3 TetO112, leu2::LEU2tetR-GFP, TRP1::MET3promoter-Cdc20*
BR779	*MAT**a**, eco1-1, SCC3 R1043L, ura3::3XURA3 TetO112, his3::HIS3tetR-GFP, TRP1::MET3promoter-Cdc20*
BR780	*MATα, ECO1 WT-NatMX, PDS5 P89S, ura3::3XURA3 TetO112, leu2::LEU2tetR-GFP, Scc3-HA3::HIS3, ADE2, TRP1::MET3promoter-Cdc20*
BR781	*MAT**a**, eco1-1, PDS5 P89S, ura3::3XURA3 TetO112, his3::HIS3tetR-GFP, TRP1::MET3promoter-Cdc20*
BR782	*MAT**a**, eco1-1, smc3Δ::HIS3, scc1Δ::KanMx, ura3::SCC1promoter-SMC3-TEV3-SCC1::URA3*
BR783	*MATα, eco1-1, SMC3 G110W, ura3::3XURA3 tetO112, his3::HIS3tetR-GFP, ADE2*
BR784	*MAT**a**, eco1-1, SCC3 R1043L, ura3::3XURA3 tetO112, his3::HIS3tetR-GFP, PDS1-MYC18::TRP1*
BR785	*MAT**a**, eco1-1, PDS5 P89S, ura3::3XURA3 tetO112, his3::HIS3tetR-GFP, PDS1-MYC18::TRP1*
BR786	*MATα, SMC1-HA6::HIS3*
BR787	*MAT**a**, eco1-1, KAN-pGal1-10-SMC3, leu2::SMC3 WT (Tagless)::LEU2*
BR788	*MAT**a**, eco1-1, KAN-pGal1-10-SMC3, leu2::SMC3 G110W (Tagless)::LEU2*
K699	*MAT**a**, ade2-1, trp1-1, can1-100, leu2-3,112, his3-11,15, ura3, GAL, psi+*
K9538	*MAT**a**, eco1-1, ura3::3XURA3 tetO112, his3::HIS3tetR-GFP, PDS1-MYC18::TRP1*
K9539	*MATα, eco1-1, ura3::3XURA3 tetO112, his3::HIS3tetR-GFP, ADE2*
K15721	*MAT**a**, WPL1-MYC18::URA3*
K16297	*MAT**a**, eco1-1*
K23308	*C. glabrata, MAT**a**, SCC1PK9::NatMX*

Table S2 - Related to Figures 1, 2, 4, 5, S2, S4 and S5
Yeast strains used in this study
All strains are derivatives of K699.

Supplemental Experimental Procedures

Yeast genetics

All yeast strains are derivatives of W303 (K699). The genetic screen for suppressors of the *eco1-1* temperature sensitive allele was in essence performed as described (Rowland et al., 2009), with the following exceptions. 250 parental independent isolates of K9538 (*MAT**a***, *eco1-1*, *TRP1*, *leu2*, etc.) and 250 independent isolates of K9539 (*MATα*, *eco1-1*, *ADE2*, *leu2*, etc.) were streaked at the non-permissive temperature on YEPD plates, and no more than one suppressor per parental clone was isolated. Each suppressor was subsequently submitted to complementation group analysis by crossing the strains to strains harbouring recessive suppressor mutations in *WAPL*, *PDS5*, *SMC3* or *SCC3*, and to a parental clone. When a suppressor failed to allow growth at the non-permissive temperature in all these diploid backgrounds, the suppressor was considered to belong to a novel complementation group, and genomic DNAs of these suppressor strains was submitted to deep sequencing. The sequencing reads were compared to genomic DNA of a parental clone. Only those mutations that were found in all reads of a suppressor DNA, but were absent in the parental DNA were selected. This yielded the responsible mutations in *SMC1*, and no further mutations. Each suppressor was subsequently backcrossed and verified by linkage analysis.

Yeast microscopy

For GFP dot assays, cells were grown in SC medium lacking methionine at 23°C and synchronized in G1 with alpha-factor. Then cells were placed to 30°C to inactivate Eco1 and medium was replaced by YEP supplemented with 2 mM methionine to arrest cells in metaphase (the *CDC20* gene is under control of the *MET3* promoter). 120 minutes after alpha-factor release, cells were harvested and fixed in ice cold 100% ethanol. For Anchor away assays, cells were synchronized in G1 by 5 µg/ml alpha-factor. Then Eco1-Aid was inactivated by addition of 500 µM of synthetic Auxin (1-Naphtaleneacetic acid). Cells were released in the presence of Auxin and Nocodazole (15 µg/ml). Rapamycin (1 µg/ml) was added when all cells were big-budded (after ± 2 hours) to 'anchor-away' Scc1-GFP-FRB (Lopez-Serra et al., 2013). Cells were harvested at indicated time-points by centrifugation and fixed by addition of ice cold 100% ethanol. Cells were mounted on a 1% agarose pad containing 0.1 µg/ml 4',6-diamidino-2-phenylindole (DAPI) and imaged on a DeltaVision Elite System (Applied Precision) using a 100x/1.4 objective and a CoolSNAP HQ2 Camera. Indirect immunofluorescence was performed on formaldehyde-fixed cells to detect PK-tagged Smc3. The following antibodies were used: mouse anti-PK (1:500 (Serotec, MCA1360)) and anti-mouse Alexafluor488 (Invitrogen). Images were acquired on the DeltaVision Elite System.

Calibrated ChIP-seq

Experiments were performed as described (Hu et al., 2015), with the exception that experimental and calibration cells were mixed before fixation. Briefly, 12 OD_{600} units of exponentially growing *S. cerevisiae* were mixed with 5 OD_{600} units of exponentially growing *C. glabrata*. Cells were fixed with a formaldehyde solution for 30 minutes and the reaction was quenched by incubating with glycine for 5 minutes. Fixed cells were harvested, washed and re-suspended in 300 µl of ChIP lysis buffer. Cells were mixed with glass beads and disrupted by FastPrep®-24 (MP Biomedicals, USA). The entire lysis was collected and sonicated for 35 minutes. Cell debris were removed by centrifugation and supernatants were adjusted to a final volume of 1ml with ChIP lysis buffer. Extracts were pre-cleared with 30 µl of Protein G Dynabeads (Invitrogen). 80 µl of supernatant was taken as whole cell extract (W) and stored at -20°C. 5 µg of anti-PK antibody (Bio-Rad) and 50 µl of Protein G Dynabeads (Invitrogen) were used for immunoprecipitation (overnight and 2 hours, rotation at 4°C). Beads were subsequently washed and immunoprecipitated chromatin was eluted by incubation of beads with 120 µl of TES buffer at 65°C for 15 minutes. The supernatants were collected and termed the IP sample. The whole cell extract sample (W) was mixed with 40 µl of TES3 buffer. Both samples were decrosslinked at 65°C overnight. RNA was degraded by incubating with RNAse A (Roche) and protein was removed by incubation with Proteinase K (Roche). DNA was purified using ChIP DNA Clean & Concentrator kit (Zymo Research, USA). For each sample, a

sequencing library was constructed using NEBNext® Fast DNA Library Prep Set for Ion Torrent™ Kit (NEB, USA) by following the manufacturers' protocol. Libraries with different barcodes were pooled together and loaded onto the Ion PI™ Chip v2 BC using the Ion Chef™ Instrument (Life Technologies, USA). Library sequencing was carried out on the Ion Torrent Proton and processed on the Galaxy platform. Occupancy ratios OR were derived from the formula OR = (IPsac/Wsac) / (IPcan/Wcan) where each parameter corresponds to the number of reads uniquely aligned to *S. cerevisiae* from IP (IPsac) or W (Wsac) samples and to *C. glabrata* from the same IP (IPcan) or W (Wcan) samples. Each data set was then normalized to a million of reads, calibrated with its respective OR and visualized on IGB. To generate an average chromosome, the chromosomes were aligned according to the CDEIII element and the number of reads at each base pair away from the CDEIII was then averaged.

Western blotting

Yeast strains are grown till OD_{600} 0.8. Cells are pelleted at 4000 rpm for 10 minutes at 4°C and washed in 20 ml cold H_2O. Cells were re-suspended in 0.5 ml of lysis buffer consisting of 25 mM HEPES pH 8.0, 2 mM $MgCl_2$, 100 µM EDTA, 500 µM EGTA, 1% NP-40, 150 mM KCl, 15% glycerol, protease inhibitor cocktail set IV (Calbiochem) and phosphatase inhibitors. Lysates were incubated on ice for 20 minutes. Glass beads are added to the lysates in 1:1 ratio and samples were shaken in a beads beater for 3 minutes. Extracts were centrifuged at 14000 rpm at 4°C for 10 minutes and supernatant was collected and frozen at -80°C. Western Blots were performed using the following antibodies: HA (Covance, 16B12), Myc (Millipore, 06-549), PK (Serotec, MCA1360), Pgk1 (Millipore, 19-101), SMC1A (Bethyl, A300-055A) Sororin (Abnova, B01p). All antibodies were used at 1:1000 dilution, except the antibody against Pgk1 was used 1:30,000). We used the following secondary antibodies: Goat anti-Rabbit-PO and Goat anti-Mouse-PO (DAKO), both at 1:600 dilution.

Recombinant protein expression

SMC1, *SMC3* and the last 351 base-pairs of *SCC1* DNA sequences of the *S. cerevisiae* genes were amplified by PCR and cloned using Ligation Independent Cloning (LIC) into the pFastBac-NKI LIC vectors (Luna-Vargas et al., 2011), resulting in constructs encoding N-terminal tags of HIS_6-Smc1 (NKI-LIC vector 2.13), StrepII-Smc3 (NKI-LIC vector 2.9) and StrepII-Scc1-C (the last 115 amino-acids of Scc1) (NKI-vector 2.13). Baculoviruses were prepared according to the BAC-to-BAC procedure (Invitrogen) and viruses were amplified twice to obtain high-titer viruses. All proteins were expressed in Sf9 insect cells (Invitrogen). HIS_6-Smc1 and StrepII-Scc1-C were co-expressed by adding equal volumes of viruses, while StrepII-Smc3 was expressed alone. Typically 8 flasks of 500 ml Sf9 suspension culture at a density of 2 x 10^6 cells/ml were infected with 1000 µl of P1 virus stock per flask and cells were harvested 72 hours after infection. Cells were harvested by centrifugation (15 minutes at 1200 rpm) and cell pellets were stored at -20°C.

Protein purification

Proteins in essence were purified as described (Haering et al., 2002). Briefly, frozen cell pellets were thawed in ice and lysed with lysis buffer consisting of 50 mM Tris pH 8.0, 10 mM KCl, 1.5 mM $MgCl_2$, 200 µM PMSF, 8 mg/ml DNase I (Roche) and protease inhibitor cocktail EDTA-free (Roche). Lysates were sonicated with 30% output for 80 seconds. Nuclei were separated from the cytosolic fraction by centrifugation at 5000g for 10 minutes. Nuclei were broken by re-suspending them in lysis buffer with increasing concentrations of NaCl until 500 mM final concentration. Cytosolic and nuclear extracts were combined together and cleared by centrifugation at 21,000 rpm at 4°C for 1 hour. For the purification of HIS_6-Smc1 and StrepII-Scc1-C, 5 mM imidazole was added to the soluble extracts and incubated with equilibrated Ni-NTA agarose beads (Qiagen). After 2 hours, the beads were washed 3 times, 10 column volumes, with washing buffers consisting of 50 mM Tris pH 8.0, 10 mM KCl, 1.5 mM $MgCl_2$, 500 mM NaCl and 5 mM imidazole, then with the same buffer but with 250 mM NaCl and finally with 100 mM NaCl and 20 mM imidazole. Proteins were eluted with 50 mM Tris pH 8.0, 10 mM KCl, 1.5 mM $MgCl_2$, 100 mM NaCl and 200 mM imidazole. The eluates were pooled and concentrated with Amicon Ultra-15 centrifugal filters (Millipore). Concentrated eluates were incubated with equilibrated *Strep*-Tactin superflow plus beads

(Qiagen) for 2 hours. Beads were washed 4 times, 3 column volumes, with 50 mM Tris pH 8.0, 10 mM KCl, 1.5 mM $MgCl_2$, 250 mM NaCl. Bound proteins were then eluted with the same buffer supplemented with 5 mM *d*-Desthiobiotin (Sigma) and 10% glycerol. Eluates were combined, concentrated and snap-frozen in liquid nitrogen prior to storing at -80°C. For StrepII-Smc3 purification, proteins were purified and eluted similar to the Strep purification protocol used for HIS_6-Smc1 and StrepII-Scc1-C. After elution, eluates were concentrated and applied to the Enrich SEC 650 10X300 size-exclusion column (Bio Rad) using 25 mM TRIS-HCl pH 8.0, 250 mM NaCl, 10 mM $MgCl_2$ and 10% glycerol. Fractions containing StrepII-Smc3 were pooled together, concentrated and snap-frozen in liquid nitrogen prior to storing at -80°C.

ATPase assays

Co-purified HIS_6-Smc1+ StrepII-Scc1-C proteins were mixed with StrepII-Smc3 at a final concentration of 2 µM in 20 mM TRIS-HCl pH 8.0, 150 mM NaCl and 10 mM $MgCl_2$. Mixed proteins were incubated with 30 µM cold ATP spiked with 1 mCi/ml [γ-^{32}P]-ATP (Perkin Elmer) at 30°C in a total volume of 10 µl. At indicated time points 1 µl of the reaction was spotted onto PEI-cellulose sheets (Merck). Thin-layer chromatography (TLC) was used to separate the reaction products using 1 M KH_2PO_4 pH 3.8 as running buffer. The sheets were dried for 30 minutes and analyzed using a phosphorimager. The quantified levels of released P_i were used to measure the percentages of hydrolyzed ATP.

Cohesin labelling & Microscale thermophoresis (MST)

Samples were labelled by incubating 1.5 μM of HIS_6-Smc1+ StrepII-Scc1-C and StrepII-Smc3 with freshly prepared TCEP in 50 mM TRIS-HCl pH 8.0, 150 mM NaCl and 1 mM $MgCl_2$ for 1 hour on ice. TCEP was removed by using a PD-10 column (GE Healthcare). The protein complex was mixed with five molar equivalents of DY-547P1 maleimide (Dyomics) and incubated at 4°C for 1 hour. Excess dye was removed using a PD-10 column equilibrated with 50 mM Tris-HCl pH 8.0, 150 mM NaCl, 1 mM MgCl2 and 0.05% Tween-20. Labelled samples were checked for fluorescence and snap-frozen in liquid nitrogen prior storing at -80°C. Thermophoresis measurements were performed in a Monolith NT.115 (Nanotemper) using hydrophilic capillaries (Nanotemper). 50 nM final concentration of the labelled samples was used in 50 mM TRIS-HCl pH 8.0, 150 mM NaCl, 1 mM $MgCl_2$ and 0.05% Tween-20. The samples were incubated with ATP or ADP nucleotides with concentrations ranging from 0.3 mM to 10 mM, for 30 minutes at room temperature. MST measurements were performed using 20% LED and 40% MST power. The laser on/off times were 30 and 5 seconds respectively. The fluorescence intensities for all the measurements were in the range of 600-1000 counts. The thermophoresis plus T-jump signal was used for creating the binding curves as described (Seidel et al., 2013). The non-linear fitting formula for MST measurements in Prism 6 GraphPad was used to measure the K_D values.

Scanning force microscopy

Co-purified Smc1 and Scc1-C proteins were mixed with Smc3 at a final concentration of 2.5 µM in 20 mM TRIS-HCl pH 8.0, 150 mM NaCl, 10 mM $MgCl_2$ and 1 mM ATP. After 10 minutes incubation at 30°C, mixed proteins were diluted to 10 nM and 20 µl was deposited on freshly cleaved mica. After about one minute the mica was rinsed with milli Q water and dried with filtered air. Samples were scanned in air at room temperature and humidity by tapping mode using a Nanoscope III or IV (Digital Instruments, CA) with a type E scanner and silicon probes from AppNano (Santa Clara, CA). Images were collected at 2 µm × 2 µm and flattened to remove background slope using Nanoscope software. Collected images from either wild type or mutant proteins were randomized and quantified blindly. Dimer structures were only quantified if the length of the arms of the V shape or the diameter of the ring was between 40 to 50 nm.

Immunoprecipitation

100 ml of yeast cultures were grown till OD_{600} 0.8. Cells are pelleted at 4000 rpm for 10 minutes at 4°C and washed in 20 ml cold water. Cells were re-suspended in 1 ml lysis buffer (25 mM HEPES pH 8.0, 2 mM $MgCl_2$, 100 µM EDTA, 500 µM EGTA, 1% NP-40, 150 mM KCl, 15% glycerol, protease inhibitor cocktail set IV (Calbiochem) and phosphatase inhibitors) and kept on ice for 30 minutes. Samples were shaken with

glass beads for 3 minutes and lysates were centrifuges at 14,000 rpm at 4°C for 20 minutes. 40 µl of cells extract were kept as input sample. Then the pre-cleared extracts were incubated with 40 µl protein A Dyna beads (Novex, Life technologies) coupled to 2 µg PK antibody (Serotec, MCA1360) for 90 minutes at 4°C. Beads were washed three times with wash buffer (25 mM HEPES pH 8.0 and 150 mM KCl) and boiled with 2x sample buffer at 95°C for 5 minutes. The supernatant was loaded onto SDS-PAGE gels and membranes were probed against the indicated antibodies.

Cell culture & chromosome spreads

HCT116 $p53^{-/-}$ cells were cultured at 37°C at 5% CO_2 in DMEM (Sigma), supplemented with 6% FCS (Clontech), 1% Penicillin/Streptomycin (Invitrogen) and 0.5% UltraGlutamin (Lonza). For colony formation assays, cells were seeded at 30,000 cells per well on 48-wells plates, transfected with siRNAs, and grown for 5 days. Plates were washed with PBS, fixed for 10 minutes using 96% methanol and stained with 0.25% crystal violet. Chromosome spreads were prepared 48 hours after siRNA transfection. Chromosome spreads were performed in essence as described (Haarhuis et al., 2013). Prior to harvesting, cells were treated with Nocodazole for 1 hour. Then cells were incubated in 0.075 M KCl at 37°C for 10 minutes and subsequently fixed with Methanol:Acetic Acid (3:1). The fixation procedure was repeated 3 times – the third time in the presence of 1 µg/ml DAPI. Chromosome spreads were generated by allowing a drop containing cells to fall from 30 cm height onto glass slides, and mounted using Prolong Antifade Gold (Invitrogen). Digital images of chromosome spreads were captured using a Metafer4/MSearch automated metaphase finder system (MetaSystems) equipped with an AxioImager Z2 microscope (Carl Zeiss). After scanning at 10x magnification, high-resolution images of spreads were acquired using a 'Plan-Apochromat' ×63/1.40 oil objective. Chromosome arm separation was quantified of at least 125 spreads, measuring the separation of the telomeres of the five longest chromosomes per spread using ImageJ software.

Genome editing & siRNAs

gRNAs targeting SMC1A were designed using an online CRISPR design tool (crispr.mit.edu): Forward: CACCGCCGCCTGACAAGTTGTCCAT Reverse: AAACATGGACAACTTGTCAGGCGGC. Annealed oligo's were ligated into pX330 (Addgene plasmid #42230). To induce the mutation we designed a homology directed repair oligo: CAACTGTGTGGCTCCTGGGAAACGCTTCCGGCCTATGGATAACGTGTCAGGC GGGGAGAAGACAGTGGCAGCTCTGGCCCTGCTCTTTGC as described (Li et al., 2014). The CRISPR construct and homology directed repair oligo were mixed in a 1:3 M ratio, and pBabePuro was added to the mix in a 10:1 ratio. The DNA mix was transfected using X-Treme Gene Transfection reagent (Roche) according manufacturers protocol. After 48 hours transfected cells were selected using 1 µg/ml puromycin, which was kept on the cells for 48 hours. Clones were picked after 10 days. Genomic DNA from monoclonal cell lines was sequenced using the primers: Forward: TGCTGGTAGAACACAGGGAG, and Reverse: GTGGCCTCAGTTCAGTCTCT. All siRNAs were manufactured by Dharmacon (ON-TARGETplus). For Sororin we used the SMARTpool and for SMC1 we used the following sequence: 5'-GCAGUAUGCUUGUGGCAAU-3'. Transfections were performed at a final concentration of 20 µM per siRNA using Invitrogen RNAiMAX (Life Technologies), following the manufacturer's instructions.

Supplemental References

Haarhuis, J.H.I., Elbatsh, A.M.O., van den Broek, B., Camps, D., Erkan, H., Jalink, K., Medema, R.H., and Rowland, B.D. (2013). WAPL-Mediated Removal of Cohesin Protects against Segregation Errors and Aneuploidy. Curr Biol *23*, 2071–2077.

Haering, C.H., Löwe, J., Hochwagen, A., and Nasmyth, K. (2002). Molecular architecture of SMC proteins and the yeast cohesin complex. Mol. Cell *9*, 773–788.

Hu, B., Petela, N., Kurze, A., Chan, K.L., Chapard, C., and Nasmyth, K. (2015). Biological chromodynamics: a general method for measuring protein occupancy across the genome by calibrating ChIP-seq. Nucleic Acids Res *43*, e132.

Li, K., Wang, G., Andersen, T., Zhou, P., and Pu, W.T. (2014). Optimization of genome engineering approaches with the CRISPR/Cas9 system. PLoS ONE *9*, e105779.

Lopez-Serra, L., Lengronne, A., Borges, V., Kelly, G., and Uhlmann, F. (2013). Budding yeast Wapl controls sister chromatid cohesion maintenance and chromosome condensation. Curr Biol *23*, 64–69.

Luna-Vargas, M.P.A., Christodoulou, E., Alfieri, A., van Dijk, W.J., Stadnik, M., Hibbert, R.G., Sahtoe, D.D., Clerici, M., Marco, V.D., Littler, D., et al. (2011). Enabling high-throughput ligation-independent cloning and protein expression for the family of ubiquitin specific proteases. J. Struct. Biol. *175*, 113–119.

Rowland, B.D., Roig, M.B., Nishino, T., Kurze, A., Uluocak, P., Mishra, A., Beckouët, F., Underwood, P., Metson, J., Imre, R., et al. (2009). Building Sister Chromatid Cohesion: Smc3 Acetylation Counteracts an Antiestablishment Activity. Mol. Cell *33*, 763–774.

Seidel, S.A.I., Dijkman, P.M., Lea, W.A., van den Bogaart, G., Jerabek-Willemsen, M., Lazic, A., Joseph, J.S., Srinivasan, P., Baaske, P., Simeonov, A., et al. (2013). Microscale thermophoresis quantifies biomolecular interactions under previously challenging conditions. Methods *59*, 301–315.
